# Characterization of red blood cell microcirculatory parameters using a bioimpedance microfluidic device

**DOI:** 10.1038/s41598-020-66693-4

**Published:** 2020-06-17

**Authors:** Tieying Xu, Maria A. Lizarralde-Iragorri, Jean Roman, Rasta Ghasemi, Jean-Pierre Lefèvre, Emile Martincic, Valentine Brousse, Olivier Français, Wassim El Nemer, Bruno Le Pioufle

**Affiliations:** 1 Université Paris-Saclay, ENS Paris-Saclay, CNRS, Institut d’Alembert, SATIE, F-91190 Gif sur Yvette, France; 2 Université de Paris, UMR_S1134, BIGR, Inserm, F-75015 Paris, France; 3 Institut National de Transfusion Sanguine, F-75015 Paris, France; 4 Laboratoire d’Excellence GR-Ex, F-75013 Paris, France; 5 Service de Pédiatrie Générale et Maladies Infectieuses, Hôpital Universitaire Necker Enfants Malades, F-75015 Paris, France; 6 Université Paris-Saclay, ENS Paris-Saclay, CNRS, Institut d’Alembert, F-91190 Gif sur Yvette, France; 7 Université Paris-Saclay, ENS Paris-Saclay, CNRS, PPSM, Institut d’Alembert, F-91190 Gif sur Yvette, France; 8 Centre de Nanosciences et de Nanotechnologies C2N, CNRS, Université Paris-Sud, Université Paris-Saclay, F-91120 Palaiseau, France; 9 ESYCOM, Univ Gustave Eiffel, CNRS UMR 9007, ESIEE Paris, F-77454 Marne-la-Vallee, France; 10 CNAM, F-75003 Paris, France; 11 Université Paris-Saclay, ENS Paris-Saclay, CNRS, Institut d’Alembert, LUMIN, F-91190 Gif sur Yvette, France

**Keywords:** Biosensors, Microfluidics, Biomedical engineering

## Abstract

This paper describes the use of a microfluidic device comprising channels with dimensions mimicking those of the smallest capillaries found in the human microcirculation. The device structure, associated with a pair of microelectrodes, provides a tool to electrically measure the transit time of red blood cells through fine capillaries and thus generate an electrical signature for red blood cells in the context of human erythroid genetic disorders, such as sickle cell disease or hereditary spherocytosis, in which red cell elasticity is altered. Red blood cells from healthy individuals, heated or not, and red blood cells from patients with sickle cell disease or hereditary spherocytosis where characterized at a single cell level using our device. Transit time and blockade amplitude recordings were correlated with microscopic observations, and analyzed. The link between the electrical signature and the mechanical properties of the red blood cells is discussed in the paper, with greater transit time and modified blockade amplitude for heated and pathological red blood cells as compared to those from healthy individuals. Our single cell-based methodology offers a new and complementary approach to characterize red cell mechanical properties in human disorders under flow conditions mimicking the microcirculation.

## Introduction

Red blood cells (RBCs) are the most abundant and simple type of blood cells whose main role is fulfilling the gas exchange to deliver oxygen to the tissues. Human RBCs are biconcave and flexible discs with an average diameter of 6–8 µm and a thickness of 2 µm. They lack nucleus and organelles and have a specific membrane composition and organization that enables them to deform and squeeze through the microcirculatory system^1^. However, under certain pathophysiological conditions, these properties are altered triggering changes in the deformability and survival rate of circulating RBCs, as observed in several human disorders like sickle cell disease and hereditary spherocytosis^2^.

Sickle cell disease (SCD) is a genetic hereditary disorder caused by a single point mutation in the β-globin gene, generating an abnormal hemoglobin (HbS) that polymerizes under hypoxic conditions leading to the sickling and alteration of circulating red cells^3^. The hallmarks of sickle cell disease are hemolytic anemia and painful vaso-occlusive crises because of the obstruction of fine capillaries^4,5^. In SCD, RBC properties are severely altered, with increased cellular dehydration, rigidity and fragility^6,7^. Hereditary spherocytosis (HS) is the most prevalent cause of hemolytic anemia due to genetic mutations in membrane or cytoskeletal proteins that disturb the structural and morphological stability of the RBC altering its biconcave shape and plasticity. HS RBCs are recognized by their spherical shape and extreme fragility, and are mainly trapped in the spleen, leading to decreased cell life span and resultant anemia^8^.

The alteration of RBC deformability, caused by rigidity disorders, can be approached by different methods. Bulk filtration, micropore filters, aspiration, ektacytometry^9^, optical tweezers and atomic force microscopy^10^ are some examples of such methods. Nevertheless, the fact of being relatively static techniques, with a low throughput, restricts the investigations to a limited number of factors involved in deformability and does not enable to address the process as a whole under physiological flow conditions. Microfluidics emerged as a new technological approach with promising applications in the biology field, and more particularly in hematology, to address cell behaviour in the microcirculation^11^. Microfluidic tools have been extensively used to mimic mammalian microcirculation in order to address blood cell circulation and adhesive interactions under normal and pathological conditions, such as in SCD^12–15^. Using specific microfluidic devices, the dynamic changes of the RBC shape were observed^16^, and the influence of microcapillaries dimensions was investigated^17^.

In this paper, we developed a bioimpedance-based microfluidic device to explore the impact of altered cell elasticity which is affected in the case of some diseases, on the transit time of RBCs flowing in microvascular-mimicking capillaries^18,19^. We investigated transit time and blockade amplitude of RBCs from heathy individuals and from patients with SCD or HS. We show evidence of increased transit time and modified blockade amplitude for heated and pathological red blood cells as compared to those from healthy individuals, as well as a heterogeneity among red cells regarding the two electrical parameters. The morphological observation of cells transiting through the fluidic restriction is also observed. We provide evidence that our microfluidic method is capable to discriminate and provide information on the RBC mechanical alterations.

Compared to other methods like ektacytometry that characterize the deformability of RBC populations^20,21^, our device mimicking the blood capillaries aims at monitoring single cells transiting through restrictions of physiological dimensions. Information at the single cell level can thus be achieved.

## Results

### Validation of the microfluidic device using heated RBCs

**Figure 1:**
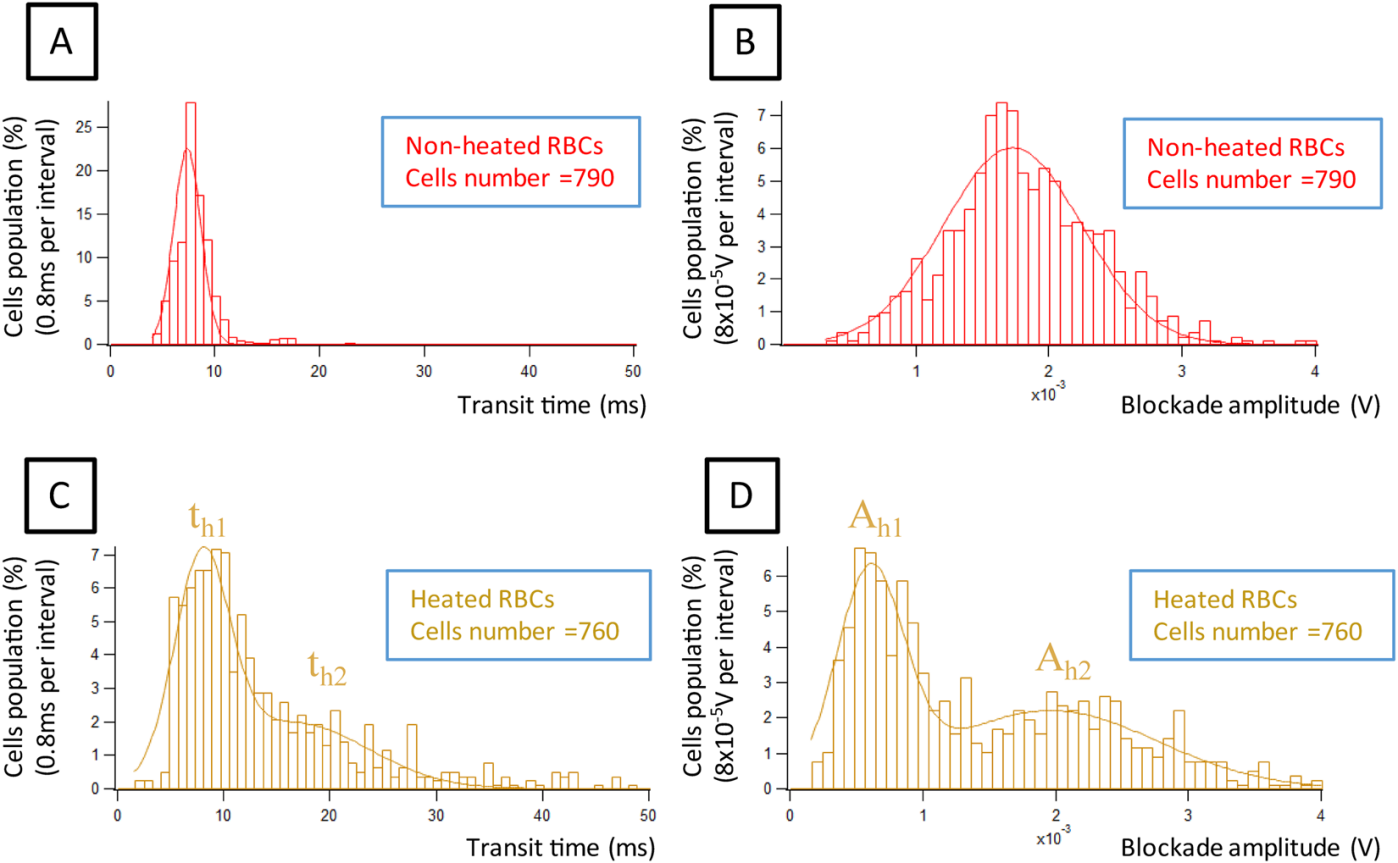
Comparison of transit time and blockade amplitude, in the case of non-heated RBCs (n = 790) (graphs **A,B**), and heated RBCs (n = 760) (graphs **C,D**). The rigidified heated RBCs present a longer transit time. The blockade amplitude is smaller for a part of the heated RBCs due to dehydration and volume decrease. Resolution is 0.8 ms for the transit time, and 8 × 10^−5^ V for the blockade amplitude.

To determine if the device enables to discriminate RBCs based on their capacity to deform, we first analyzed fresh RBCs from healthy individuals under native or heated conditions, as heat is known to drive a loss of RBC elasticity. RBC suspensions from 3 healthy donors were prepared and split into two, with one half of the suspension undergoing a 5 min incubation time at 60 °C. We determined the transit time and the blockade amplitude of control RBCs in our device. We recorded these values for 790 native RBCs (from the 3 healthy donors) and found a Gaussian distribution for both parameters, with a main transit time of 7.39 ± 0.09 ms and a main blockade amplitude of (1.73 ± 0.02) × 10^−3^ V (Fig. 1A,B and Supplementary Table S1). Similarly, we recorded these values for 760 heated RBCs and found increased transit time (mean $\overline{{t}_{h}}$ = 16 ms) (Fig. 1A,C) and decreased blockade amplitude (mean $\overline{{A}_{h}}$ = 1.45 × 10^−3^ V) (Fig. 1B,D) as compared to native RBCs, indicating that rigid and less deformable RBCs are globally delayed in the system. There was some heterogeneity among the 3 donor samples, with a variance of 0.018 ms^2^ for transit time and 0.011 × 10^−6^ V^2^ for blockade amplitude (see Supplementary Fig. S1). However, despite this variability, the parameters showed the same trend, with increasing transit time and decreasing blockade amplitude in the case of heated RBCs (see Supplementary Fig. S2. for histograms of one donor RBCs).

We found that transit time and blockade amplitude distributions were more spread for heated than for native RBCs, with heated RBCs showing bimodal distribution of both parameters. For transit time, heated RBCs displayed two maxima (t_h1_ = 8 ms and t_h2_ = 16.5 ms) (Supplementary Table S1), suggesting that the heating protocol affects RBCs differentially, which was not surprising as the RBC population in the circulation is heterogenous in terms of age and other intrinsic properties. Considering the distribution of the blockade amplitude, heated RBCs presented globally lower values than non-heated RBCs (see Fig. 1B,D). Indeed, 63.3% of heated RBCs had smaller blockade amplitude than the peak value (µ) of non-heated RBCs distribution (1.7 × 10^−3^ V). This could be explained by the reduced RBC volume after the heating step, with a less efficient electrical blockade in the restriction zone by smaller RBCs. To confirm the decrease in cell volume after the heating step, we measured RBC diameter using optical microscopy. As expected, heated RBCs were spherical and had a smaller diameter as compared to non-heated RBCs (Supplementary Fig. S3)^22^. Nevertheless, the blockade amplitude of the other subpopulation was greater than for native RBCs (1.96 × 10^−3^ V) confirming that rigid RBCs had higher blockade amplitude than deformable ones.

The difference between heated and non-heated RBCs was +0.6 ms in term of transit time, and −1.1 × 10^−3^ V in term of blockade amplitude for the main peak.

The phase measurement was also performed. A decrease of phase shift for one subpopulation of heated RBCs (−0.05 rad) was noticed (see Supplementary Fig. S4).

### Electrical signature of RBCs from patients with hereditary spherocytosis

After validating our system with rigid RBCs generated *in vitro* by a heating step, we used the biochip to determine the electrical signature of RBCs from patients with hereditary spherocytosis (HS). HS is a red cell membrane disorder that affects the protein-protein interactions between the RBC cytoskeleton and the membrane, generating spherical and poorly deformable RBCs.

**Figure 2:**
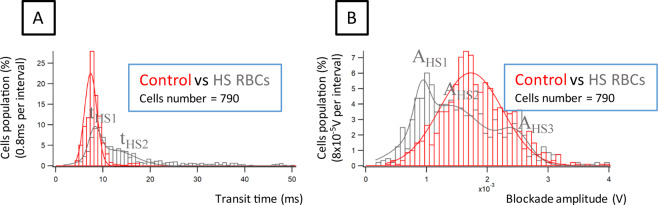
(**A**) Transit time of control and HS RBCs. For HS RBCs, the transit time is more dispersed with a longer transit time for a part of the cell population; (**B**) Blockade amplitude of control and HS RBCs. Two main subpopulations are observed when examining the blockade amplitude (Supplementary Table S2). Transit time resolution of 0.8 ms and a blockade amplitude resolution of 8 × 10^−5^ V were chosen. 790 RBCs were analyzed from 3 healthy donors and 3 HS patients.

RBCs from 3 HS patients were analyzed and compared to control RBCs. There was some heterogeneity among the 3 patient samples but the parameters showed the same trend, with increasing transit time (Fig. 2A) and decreasing blockade amplitude (Fig. 2B) as compared to control native RBCs (see Supplementary Fig. S5. for histograms of one HS patient). The difference between HS and control RBCs was +0.9 ms for transit time, and −0.8 × 10^−3^ V for blockade amplitude. Transit time was found to be longer for HS than control RBCs, with a broader distribution exhibiting at least two subpopulations, indicating a heterogeneous deformability within HS RBCs (see Fig. 2A). Considering the blockade amplitude, at least two main subpopulations of HS RBCs were observed: one subpopulation with a blockade amplitude that was smaller than the one of control RBCs and another subpopulation with higher blockade amplitudes (Fig. 2B). These results were similar to the results obtained with heated RBCs which was not surprising as heating RBCs is known to drive a spherocytosis-like phenotype. This indicated that our system was efficient to stratify RBCs based on their transit time and blockade amplitude, both characteristics being affected by the capacity of the cells to deform when passing the mechanical restriction.

Comparing the phase shift between HS and control RBCs, a decrease of −0.02 rad in the case of HS RBCs was noticed (see Supplementary Fig. S6).

### Electrical signature of RBCs from patients with sickle cell disease

**Figure 3:**
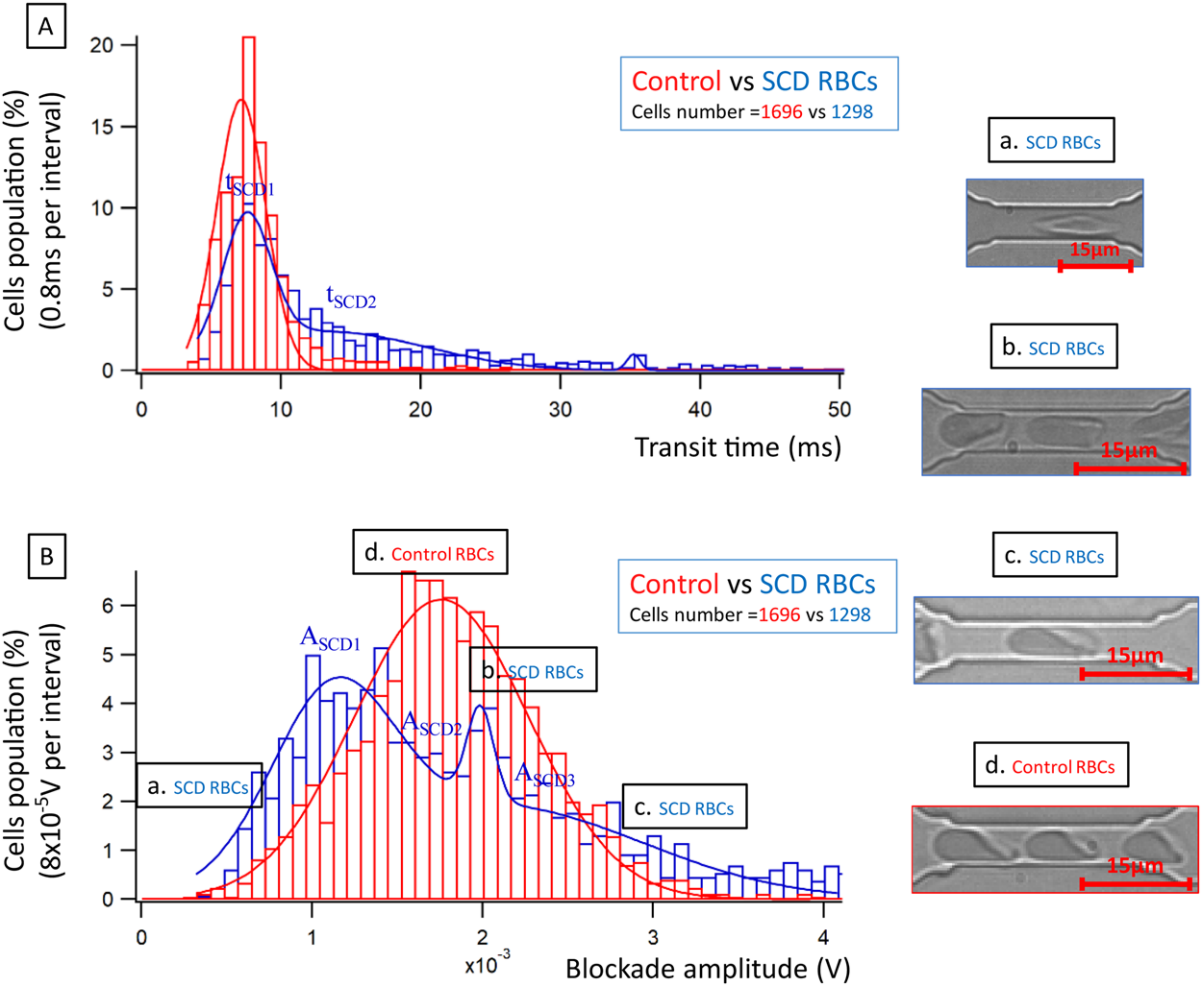
(**A**) Comparison of average transit time of control and SCD RBCs. For SCD RBCs, the transit time is more dispersed with a longer transit time for a part of the cell population; (**B**) Comparison of average blockade amplitude of control and SCD RBCs. Three main populations are observed when examining the blockade amplitude (Supplementary Table S3). The transit time resolution is 0.8 ms and the blockade amplitude resolution is 8 × 10^−5^ V. On these graphs data was collected from 1696 control RBCs coming from 6 healthy donors, and 1298 SCD RBCs coming from 5 patients. Images a, b and c (graph B) correspond to subpopulations of SCD RBCs, while image d corresponds to control RBCs.

Finally, we used the biochip to analyze RBCs from patients with SCD. In SCD, RBCs of a given patient are very heterogeneous in terms of size, shape, hydration status and deformability. We analyzed RBCs from 6 healthy donors and 5 SCD patients. Similar to heated and HS RBCs, there was a variability among SCD RBCs, but the transit time was increased (Fig. 3A) and the blockade amplitude was decreased (Fig. 3B) for all SCD samples when compared to control (see Supplementary Fig. S7. for histograms of one SCD patient). Transit time of SCD RBCs was greater and showed a broader spectrum compared to control RBCs (Fig. 3A). For blockade amplitude, there was two main peaks for SCD RBCs, with one subpopulation showing values smaller than those of control RBCs (first peak on the left of Fig. 3B). One possibility residing behind this observation is the shape and the orientation in the flow of a category of SCD RBCs called irreversibly sickled cells (ISCs), as we have previously reported^23^. Indeed, contrary to control RBCs that have a biconcave shape, ISCs are elongated cells that can flow through capillaries with almost no contact with the capillary wall (Fig. 3Ba). A second subpopulation of SCD RBCs showed higher blockade amplitudes compared to control RBCs. This indicates the presence of a significant proportion of RBCs with decreased deformability, which is something expected and described for SCD RBCs that present high numbers of dehydrated dense cells.

The phase measurement was also performed. A decrease of −0.03 rad in the case of SCD RBCs was noticed (see Supplementary Fig. S8). This might be due to the membrane damages inducing an alteration of its insulating properties, thus increasing the in-phase component of the current corresponding to the leakage through the cell membrane.

In addition to the electrical monitoring, we performed experiments with a higher hematocrit (30% instead of 2%) with the same value of depressurization (250 mbar) in order to optically record several RBCs passing simultaneously within one restriction. Morphological observations were performed (for more information, see Supplementary Fig. S9). Control RBCs showed the well-known parachute shape (Fig. 3Bd)^24^. Different shapes of SCD RBCs were observed; in addition to the elongated shape mentioned above for ISCs (Fig. 3Ba), there were cells with higher contact surface area than control RBCs (Fig. 3B.b,c), which could explain the increase of the blockade amplitude that was observed for a subpopulation of SCD RBCs. In some cases, smaller cell velocity was observed within the restriction, sometimes conducting to occlusion.

## Discussion

A microfluidic device, simulating the smallest capillaries of blood microcirculation, is proposed in this paper to characterize electrically the transit of RBCs. A bioimpedance-based approach enabled to discriminate RBCs based on their transit time and amplitude blockade on a single cell level.

The device is built on a biochip that we have previously developed to address the impact of repeated mechanical stress on the ability of RBCs to resist haemolysis^23^. The original biochip was designed to process millions of RBCs in a 15–20 min frame thanks to 8 restriction units each composed of 24 parallel channels with 10 serial restrictions of 5 µm in each channel. In the current study, we added a pair of microelectrodes to one of the 1920 restrictions (8 × 24 × 10) of the biochip in order to collect the electrical signature of the flowing cells. This information, combined with the haemolysis values, is a step forward to study RBC properties under microcirculatory conditions in a comprehensive manner.

Although the number of cells that were electrically characterized by the device was relatively low compared to the high number of RBCs in the circulation, it was sufficient to achieve statistical relevance.

The next step would be to implement the device with more microelectrodes and to scale up the number of processed cells per time unit in order to process larger cell numbers to envision the use of such method and device for advanced medical diagnostic.

In 2013, a comparable technique allowing the electrical monitoring of cells transiting in a microchannel was reported^19^. In this previous study, where the loss of RBC elasticity was induced either by heating or by fixation with glutaraldehyde, the authors showed that rigid RBCs had longer transit time in microcapillaries. Our study confirms these findings with heated RBCs and, more importantly, further extends them by exploring native pathological RBCs, with no *in vitro* manipulation prior to the perfusion in the microfluidic device. Analysing such pathological RBCs we show that the device is adapted to generate electrical signatures of RBCs in the context of HS and SCD, both pathologies being characterized by poorly deformable RBCs^25,26^, and to characterize the distribution of RBCs in a cell suspension regarding the transit time and the blockade amplitude.

Although affected by different molecular defects, HS and SCD RBCs share the common feature of being heterogenous in terms of shape, morphology and mechanical properties^25–27^. In HS, reduced RBC deformability results from mutations altering protein-protein interactions in the skeleton underlying the cytoplasmic membrane while in SCD it is secondary to a mutation in the β globin chain driving haemoglobin polymerization and resulting in red cell sickling and dehydration. The loss of RBC deformability contributes to clinical manifestations in both pathologies, such as haemolytic anaemia, because of RBC lysis in the circulation, splenic sequestration, and the vaso-occlusive crisis in the case of SCD^7^. The proportion of poorly deformable RBCs in SCD is variable among patients, and the percentage of dense RBCs is a severity marker as these cells can initiate or contribute to vascular occlusion and subsequent tissue ischemia^28^. The microfluidic device developed in this study was sensitive enough to show this heterogeneity among the analyzed RBCs, despite two different molecular origins, and to quantify the proportions of each subpopulation regarding the transit time and the amplitude blockade. Considering the relationship between the proportions of poorly deformable RBCs and clinical manifestation in SCD, this opens good perspectives for the use of our device, or an improved high throughput version of it, for drug screening approaches aiming at identifying new molecules that positively impact RBC deformability, as well as for testing the impact of therapeutical strategies on this parameter during pre-clinical and clinical studies with SCD patients.

The comparison between control RBCs from healthy donors and heated RBCs, SCD RBCs and HS RBCs was performed on the chart transit time vs blockade amplitude. The graph, shown in Supplementary Fig. S10. shows that distinct area corresponds to the different pathologies (HS RBCs, SCD RBCs), compared to control or heated RBCs. Even though broader analysis including more cells from more patients is still required, the current results open the perspective of using such microfluidic approach for the diagnosis of hereditary diseases affecting RBC properties.

Higher capabilities of automated treatment of the data should also be developed to envision the higher throughput of such electrical monitoring.

Our methodology, relying on the use of the microfluidic chip, offers a new and efficient approach to characterize the RBC physiological state, and thus qualify the deformability alteration that occurs in the case of hereditary spherocytosis and sickle cell disease, or potentially in any other disorder that affects RBC elasticity. In a future study, we aim at developing a more complex device to enable monitoring electrically the passage of cells through several consecutive restrictions.

## Methods

**Figure 4:**
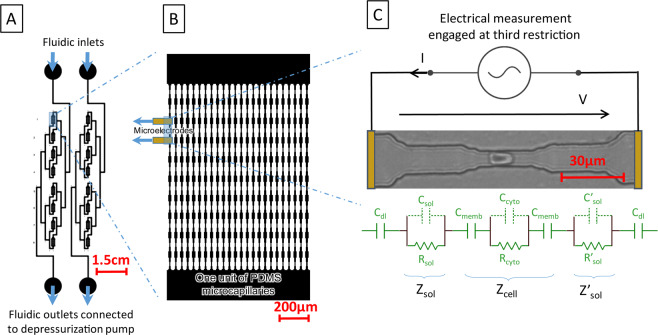
(**A**) The blood samples are deposited at fluidic inlets while outlets are depressurized, using pressure controller. Two channel networks are integrated on the same chip. The sample is distributed in parallel to eight units. (**B**) One unit is represented, 24 channels in parallel, presenting 10 successive restrictions. Electrodes are connected with excitation generator via dedicated printed circuit board containing gold spring needles. (**C**) Equivalent electrical model for electrical monitoring of RBC passing through the third restriction of extern branch at the first unit, electrodes are connected to the excitation generator via dedicated printed circuit board.

The alteration of RBC deformability, interrelated with sickle cell disease, is considered using electrical signature of the cell passing through a microfluidic restriction. To mimic blood flow within organs and the mechanical stresses endured by RBCs, a microcapillary network, reproducing physiological conditions, is proposed (see Fig. 4A). First, the blood capillary dimensions are reproduced using PDMS casting technology on a multi-layer SU8 mold that presents restrictions with dimensions smaller than RBC (see Fig. 4B). As shown in Fig. 4C, a single restriction is arrayed in the microfluidic device in order to achieve a consistent flow, that allows the sample collection after mechanical treatment in the series of mechanical constrictions^23^. The whole device contains 8 units in parallel. Each unit contains 24 channels with 10 restrictions in series. Each restriction is based on 3 gradually reduced sections: starting with a section of 20 µm × 2 µm with a length of 20 µm, the RBC is flattened on the substrate; a second section of 10 µm × 2 µm enables to center the RBCs within the microchannel; the third section of 5 µm × 2 µm squeezes the RBCs within a 30 µm long section. There, the RBC is elongated and submitted to a mechanical stress. The RBC flows out the mimicking microcapillary, recovering its initial shape across gradually increasing sections^23^.

The cell transit signature is monitored electrically thanks to a pair of electrodes disposed at both sides of a restriction. In our case, the pair of electrodes is placed below the third restriction of the outer microchannel (see Fig. 4B) The electrodes that are microfabricated using conventional clean room technology on a quartz substrate need to be aligned with the PDMS channels. In order to facilitate such alignment, we chose a non-permanent assembly between the electrical and microfluidic levels. In order to achieve a suitable sealing between both parts, a depressurization is applied at the outlet of the channel network, while the sample is loaded at the inlet. Gas bubbling, that should appear in those depressurization conditions due to the PDMS permeability to gas, was avoided thanks to the coating of a parylene layer on the PDMS chip surface. A digital lock-in amplifier was used to determine (1) the RBC transit time and (2) the RBC blockade signal. Such information is correlated to RBC diseases and RBCs physiological state^29^ altering the RBC deformability.

The current I, collected at the electrode is modulated by the presence of the cell in the restriction. ${\rm{I}}=\frac{{\rm{V}}}{{\rm{Z}}}$, V being the applied voltage and Z the electrical impedance between both electrodes that expresses:

1 $${\rm{Z}}={{\rm{Z}}}_{{\rm{cell}}}+{{\rm{Z}}}_{{\rm{sol}}}+{{\rm{Z}}{\prime} }_{{\rm{sol}}}+2\ast {{\rm{Z}}}_{{\rm{dl}}}$$

The equivalent circuit model was used to estimate the impedance spectrum, presented in Supplementary Fig. S11., and compared with experimental measurements.

${{\rm{Z}}}_{{\rm{cell}}}$ is the complex impedance of the flowing RBC, ${{\rm{Z}}{\prime} }_{{\rm{sol}}}$ and ${{\rm{Z}}}_{{\rm{sol}}}$ are the impedance of the two side-sections of the restriction filled with the medium beside the flowing RBC.

To calculate the electrical impedance of a fluidic restriction without the presence of a cell, the geometry of the restriction, composed of several segments presenting decreasing sections, was considered. Each of those segments, having a section ${{\rm{S}}}_{{\rm{s}}}$ and a length ${{\rm{D}}}_{{\rm{s}}}$, has its own capacitance (${{\rm{C}}}_{{\rm{s}}}=\frac{{{\rm{\varepsilon }}}_{{\rm{m}}}{{\rm{S}}}_{{\rm{s}}}}{{{\rm{D}}}_{{\rm{s}}}}$) and resistance (${{\rm{R}}}_{{\rm{s}}}=\frac{1}{{{\rm{\sigma }}}_{{\rm{m}}}}\frac{{{\rm{D}}}_{{\rm{s}}}}{{{\rm{S}}}_{{\rm{s}}}}$), that has to be put in serie in order to calculate ${{\rm{C}}}_{{\rm{sol}}}$ and ${{\rm{R}}}_{{\rm{sol}}}$. ${{\rm{C}}}_{{\rm{sol}}}$ contribution to the impedance is negligible at the considered frequencies.

When RBC transit in the smallest section of the restriction, the cell capacitance ${{\rm{C}}}_{{\rm{cyto}}}$ (${{\rm{C}}}_{{\rm{cyto}}}=\frac{{{\rm{\varepsilon }}}_{{\rm{cyto}}}{\rm{S}}}{{\rm{D}}}$, negligible contribution at those frequencies) and resistance ${{\rm{R}}}_{{\rm{cyto}}}$ (${{\rm{R}}}_{{\rm{cyto}}}=\frac{1}{{{\rm{\sigma }}}_{{\rm{cyto}}}}\frac{{\rm{D}}}{S}$) are calculated using the surface S of the smallest section of the capillary. D represents the length of the cell while being in the smallest capillary, and is calculated from the cell volume which is kept constant despite the RBCs deformation. Dielectric characteristics for the cell membrane and cytoplasm were found from literature^30^.

The cell membrane, which insulates the cytoplasm from the solution, is modelled by its capacitance ${{\rm{C}}}_{{\rm{memb}}}$^30^.

As a pair of electrodes is disposed at both sides of the restriction, the double layer capacitance ${{\rm{C}}}_{{\rm{dl}}}$ (in serie with the restriction impedance) at the surface of electrodes needs also to be considered^31^:

2 $${{\rm{Z}}}_{{\rm{dl}}}=\frac{1}{{\rm{j}}\,\ast \,{{\rm{C}}}_{{\rm{dl}}}\,\ast \,{\rm{\omega }}}$$

${{\rm{C}}}_{{\rm{dl}}}$ is the double layer capacity. ${{\rm{C}}}_{{\rm{dl}}}={{\rm{C}}}_{{\rm{surf}}}\,\ast \,{\rm{S}}$, where S is the surface of considered electrode and ${{\rm{C}}}_{{\rm{surf}}}$ the surface capacitance (${{\rm{C}}}_{{\rm{surf}}}$ =10^−2^ F.m^−2^)^32^.

With these assumptions, the impedance spectrum was estimated with and without the presence of the cell in the restriction using i) finite element analysis (COMSOL Multiphysics) and ii) our analytical model (see Supplementary Fig. S11). Electrical properties of the medium used for experiments are its conductivity σ_m_ = 1.26 S/m and relative permittivity ε_m_ = 80. The RBC cytoplasm conductivity and relative permittivity were assumed to be σ_cyto_ = 0.9 S/m and ε_cyto_= 50^33^, while the RBC membrane was defined by the membrane conductivity σ_memb_= 10^−5^ S/m and the membrane relative permittivity ε_memb_= 11^33^. Three distinct zones of the impedance spectrum can be distinguished: (1) the electrode polarisation dominant zone (zone 1, f < 500 Hz), (2) the medium conduction dominant zone (zone 2, 500 Hz <f < 10^4^ Hz), and (3) a frequency band in which the device parasitic capacitance induces a low-pass filtering (f > 10^4^ Hz).

As the transit time of RBCs was measured to be at least few milliseconds, 10^4^ Hz was chosen as the working frequency for the AC excitation V and 500 kHz for the sampling frequency. An impedance variation of 11% is predicted by finite element analysis, induced by the presence of the RBC in the restriction. Using the lock-in amplifier, the RBC transit time as well as the RBC blockade signal were extracted for each cell flowing through the restriction.

**Figure 5:**
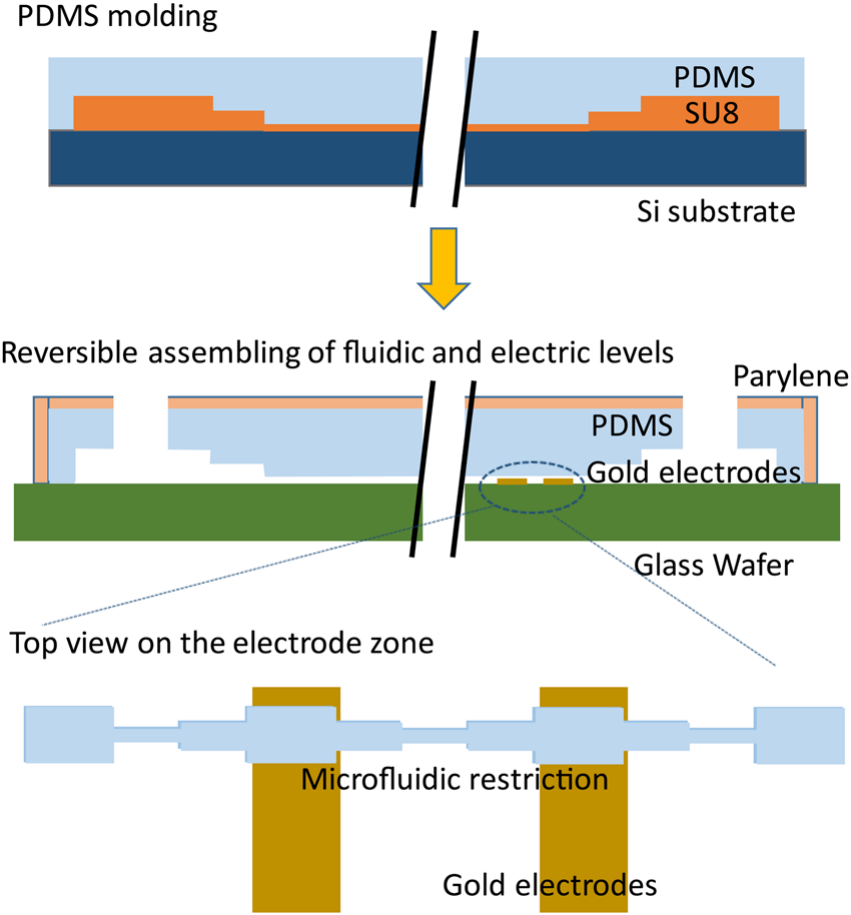
Fabrication process of reusable microfluidic device coated by a parylene layer.

The microfluidic level of the device is casted in PDMS, requiring a three-level mold made in SU8 thick resist. The electric level was made thanks to the patterning of gold electrodes on a quartz substrate. Both parts were aligned and reversibly assembled prior to experiment (see Fig. 5).

For the three-layers SU8 mold fabrication: a conventional UV lithography method was used with 3 successive types of SU8 photoresist: SU8 2002, SU8 2005 and SU8 2025 (Microchem), respectively to achieve the 2, 5 and 25 µm height of channels.

First, after cleaning in an ozone plasma cleaner during 5 min, the silicon wafer was covered by SU8 2002 (spin coating at 3000 rpm with an acceleration of 300 rpm/s² during 30 s). A soft bake was done (65 °C for 1 min, 95 °C for 2 min, 65 °C for 1 min) before UV lithography (14 s, 150 mJ/cm^2^). Then the post exposure bake was performed (65 °C for 1 min, 95 °C for 5 min, 65 °C for 1 min) followed by 2 min development (SU8 Developer Microchem) and isopropanol rinsing. 60 °C drying was then done during 15 min. This protocol was repeated for the SU8 2005 layer. To obtain the SU8 2025 layer, defining the large channels that connect the 8 units to the inlet and outlet of the device, the parameters were 1) soft bake (65 °C for 1 min, 95 °C for 7 min, 65 °C for 1 min) 2) followed by UV exposure (21 s, 150 mJ/cm^2^), 5 min for development 3) and 1 h 175 °C hard bake was finally performed.

To achieve the PDMS casting, a mixture of PDMS and curing agent was poured with a proportion of 10:1 after degassing on the SU8 mold. The reticulation was performed using 75 °C, 2 h heating. After PDMS casting, a parylene layer (thickness of 3.5 µm)^34^ was deposited in order to eliminate air bubbling in the microchannels caused by the depressurization applied at the channel outlet during the experiments.

Concerning the fabrication of the electric level: a substrate of quartz covered with a gold layer of 200 nm on a chromium layer as attachment layer (20 nm) was used. The gold electrodes patterning was achieved thanks to conventional UV lithography process. 3000 rpm^35^ spin coating of S1805 followed by soft bake (115 °C, 2 min), UV illumination through the mask (12 s, 150 mJ/cm^2^), developing, rinsing, gold etching (use of KI, 45 s) and Cr etching (15 s). The S1805 resin layer was removed using acetone.

For the reversible assembling of fluidic and electric levels of the bio-device, electric and fluidic levels were aligned manually using a low magnification binocular (5×). The sample was gently loaded at the fluid inlet, while a negative pressure was applied to the fluid outlet of the device, to induce the blood flowing within the microfluidic device.

**Figure 6:**
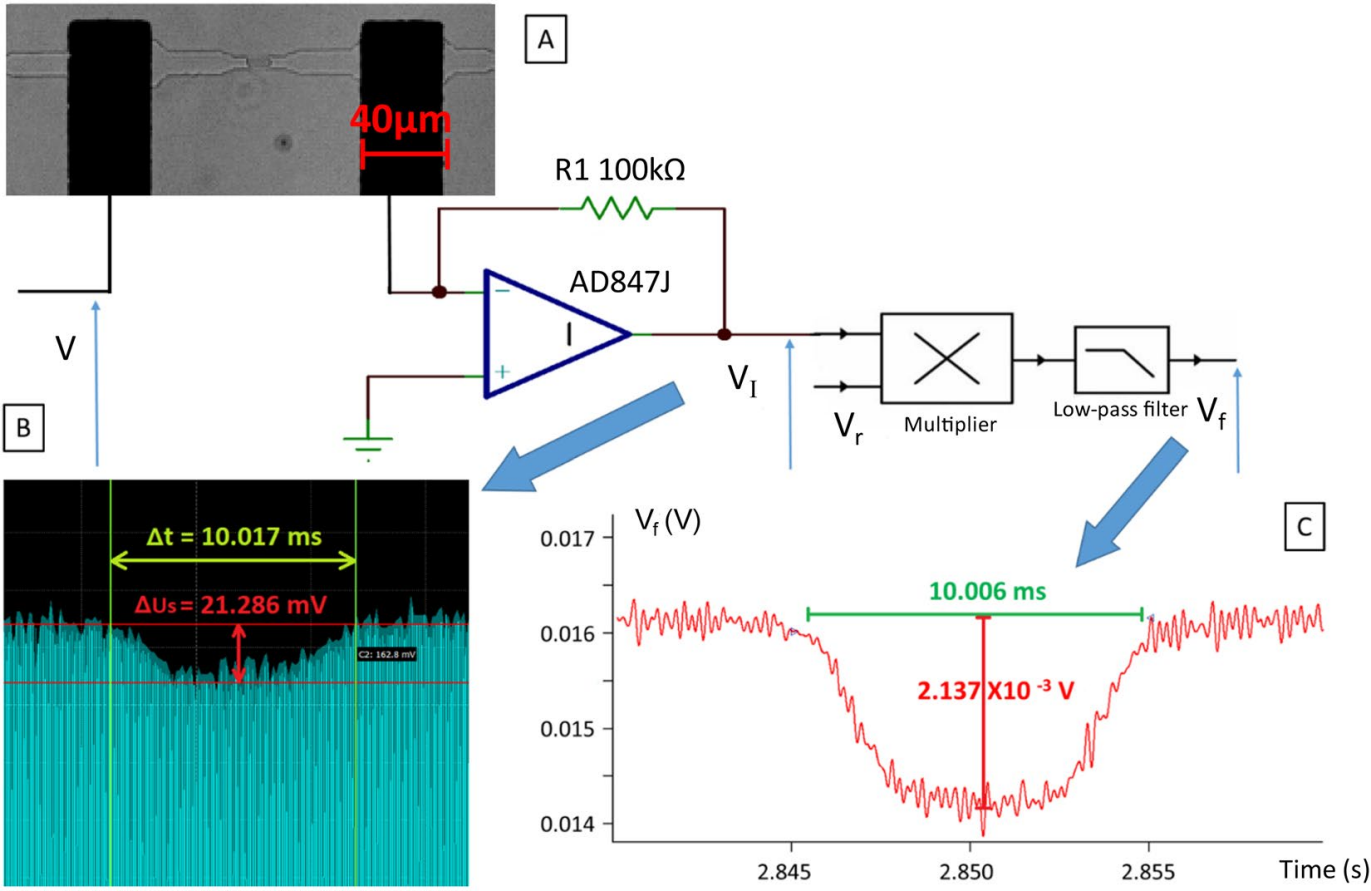
(**A**) RBC passing through a microcapillary under optic microscope; (**B**) Signal variation when RBC pass through the microcapillary; (**C**) Signal after digital lock-in amplifier.

For the electrical recording system, the electrical signal collected by the two micro-electrodes inserted within the circuit was measured and filtered thanks to a homemade circuit board (see Fig. 6A).

Using the Analog Discovery device^36^, an alternative voltage V (amplitude of 2 V, frequency of 10 kHz) was applied to the electrodes. The current I was measured with the trans-impedance amplifier, as mentioned above, with 500 kHz sampling frequency, for each cell passing through the sensing zone between the electrodes (see Fig. 6B). The signal produced by the transit of several cells in the restriction was not taken into account.

The circuit board, contains an amplifier AD847J^37^, used as a trans-impedance amplifier. The acquired signals were then digitally filtered using IGOR Pro software. A 5000 Hz low pass filter was therefore employed (order 10). Transit time, amplitude of blockade, and phase shift are extracted from this signal as explained here after (see Fig. 6C)^38^.

The signal provided by the trans-impedance amplifier expresses:

3 $${{\rm{V}}}_{{\rm{I}}}({\rm{t}})={\hat{{\rm{V}}}}_{{\rm{I}}}\,\sin (2{{\rm{\pi }}{\rm{f}}}_{{\rm{s}}}{\rm{t}}+{{\rm{\phi }}}_{{\rm{s}}})$$

where ${\hat{{\rm{V}}}}_{{\rm{I}}}$ is the amplitude of the signal, f_s_ its frequency, ${{\rm{\phi }}}_{{\rm{s}}}$ is the phase shift introduced by the presence of the cell (${{\rm{\phi }}}_{{\rm{s}}}=0$ when there is no cell within the restriction). To achieve the synchronous detection, ${{\rm{V}}}_{{\rm{I}}}({\rm{t}})$ is multiplied by the reference signal:

4 $${{\rm{V}}}_{{\rm{r}}}({\rm{t}})=\,\sin (2{{\rm{\pi }}{\rm{f}}}_{{\rm{s}}}{\rm{t}})$$

We get:

5 $${{\rm{V}}}_{{\rm{I}}}({\rm{t}}){{\rm{V}}}_{{\rm{r}}}({\rm{t}})=\frac{{\hat{{\rm{V}}}}_{{\rm{I}}}\,}{2}(\cos ({{\rm{\phi }}}_{{\rm{s}}})-\,\cos (4{\rm{\pi }}({{\rm{f}}}_{{\rm{s}}}){\rm{t}}))$$

using a ten-order low-pass filter, the $(2{{\rm{f}}}_{{\rm{s}}})$ frequency component is highly alternated, and we get a filtered signal that can be approximated to:

6 $${{\rm{V}}}_{{\rm{f}}}=\frac{{\hat{{\rm{V}}}}_{{\rm{I}}}\,}{2}\,\cos ({{\rm{\phi }}}_{{\rm{s}}})$$

finally from ${{\rm{V}}}_{{\rm{f}}}$, one can estimate i) the blockade duration Δτ which corresponds to the time duration where the signal ${{\rm{V}}}_{{\rm{f}}}$ is attenuated by the presence of the cell within the restriction, ii) the blockade amplitude $\frac{{\hat{{\rm{V}}}}_{{\rm{I}}}\,}{2}(1-\,\cos ({{\rm{\phi }}}_{{\rm{s}}}))$.

An MFCS-EZ microfluidic flow control system (Fluigent) was used to regulate the depressurization in the microfluidic biochip. The biochip was connected to the pump by 1.6 mm silicone tubing and male luer connectors and mounted on the stage of an inverted AxioObserver Z1 microscope (Zeiss) coupled with a Phantom Miro M 320 S high-speed camera in order to acquire the cells passing through the restriction unit, and in particular through the restriction zone placed between the sensing pair of electrodes. For each assay, the RBC suspension at 2% hematocrit were loaded in the input well of the biochip and a constant depressurization of 250 mBar for 20 min was exerted. This concentration leads to an average count of 165 cells in each unit (composed 24 channels), and thus an average of 6–7 cells per channel. This was confirmed by our microscope observation during experiment. As the sensing of RBC abnormal rigidity was targeted in this study, the survival rate of cells flowing through our device was out of our focus. It should be considered in further study. One advantage of our device is that it can be used several experiments. From two to three experiments were done for each sample using the same microfluidic device, in order to confirm the repeatability of results. After each experiment, the PDMS subpart of the microfluidic device was washed with distilled water followed by Tween-20 detergent solution. The lifetime of the device was limited by its PDMS subpart, due to the parylene layer degradation after the cleaning. The study was conducted in accordance with the Declaration of Helsinki and was approved by the Regional Ethics Committee (n°3215 CPP Ile de France III). Blood samples were recovered from blood tubes drawn for medical care after written informed consent. Blood samples were collected on ethylenediaminetetraacetic acid (EDTA) from 5 non-treated and non-transfused patients with sickle cell anemia (SS and Sbeta° genotypes), 3 patients with hereditary spherocytosis and from 6 healthy donors (Etablissement Français du Sang).

After 10 min centrifugation at 1500 rpm, plasma and buffy coat where eliminated from the blood samples. Three washes with CellStab (BIORAD) were done under the same centrifugation conditions, and a 2% hematocrit solution was prepared in HBSS-BSA 0.4%, 1 mM CaCl_2_, 1 mM MgCl_2_ solution. The high conductance of this solution (12.64 mS/cm, 1% F.S. to 19.99 mS/cm) facilitates to measurement of the cell passage signal through the restriction area. For heated control RBCs, the 2% hematocrit suspension was incubated at 60 °C for 5 min.

## Supplementary information

Supplementary information.

Supplementary information2.

Supplementary information3.

Supplementary information4.

Supplementary information5.

Supplementary information6.

Supplementary information7.

Supplementary information8.

Supplementary information9.

Supplementary information10.

Supplementary information11.

Supplementary information12.
