# Supplementary figures and images for: Characterization of red blood cell microcirculatory parameters using a bioimpedance microfluidic device
Source: Sci Rep. 2020 Jun 17;10:9869. doi: 10.1038/s41598-020-66693-4 (PMC7299978; doi:10.1038/s41598-020-66693-4)

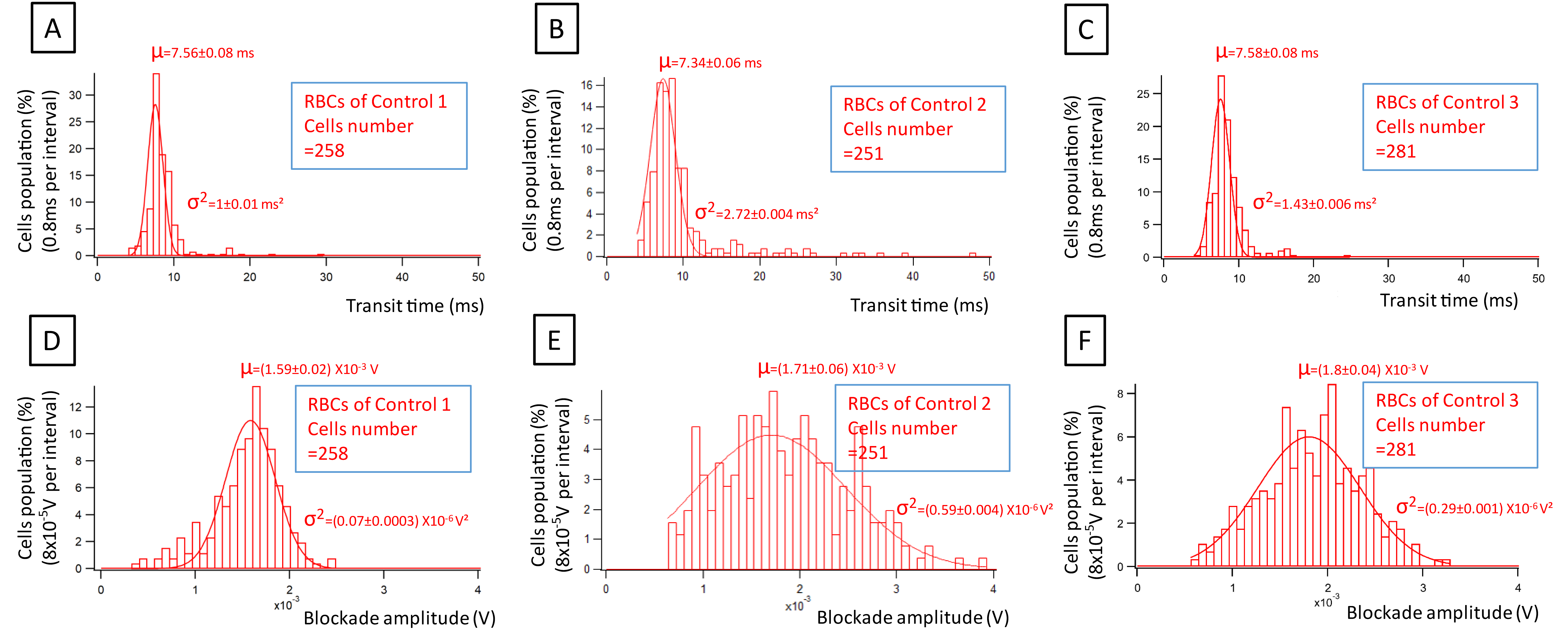

Supplement: Supplementary file 1 — Supplementary information. [file 41598_2020_66693_MOESM1_ESM.tif]

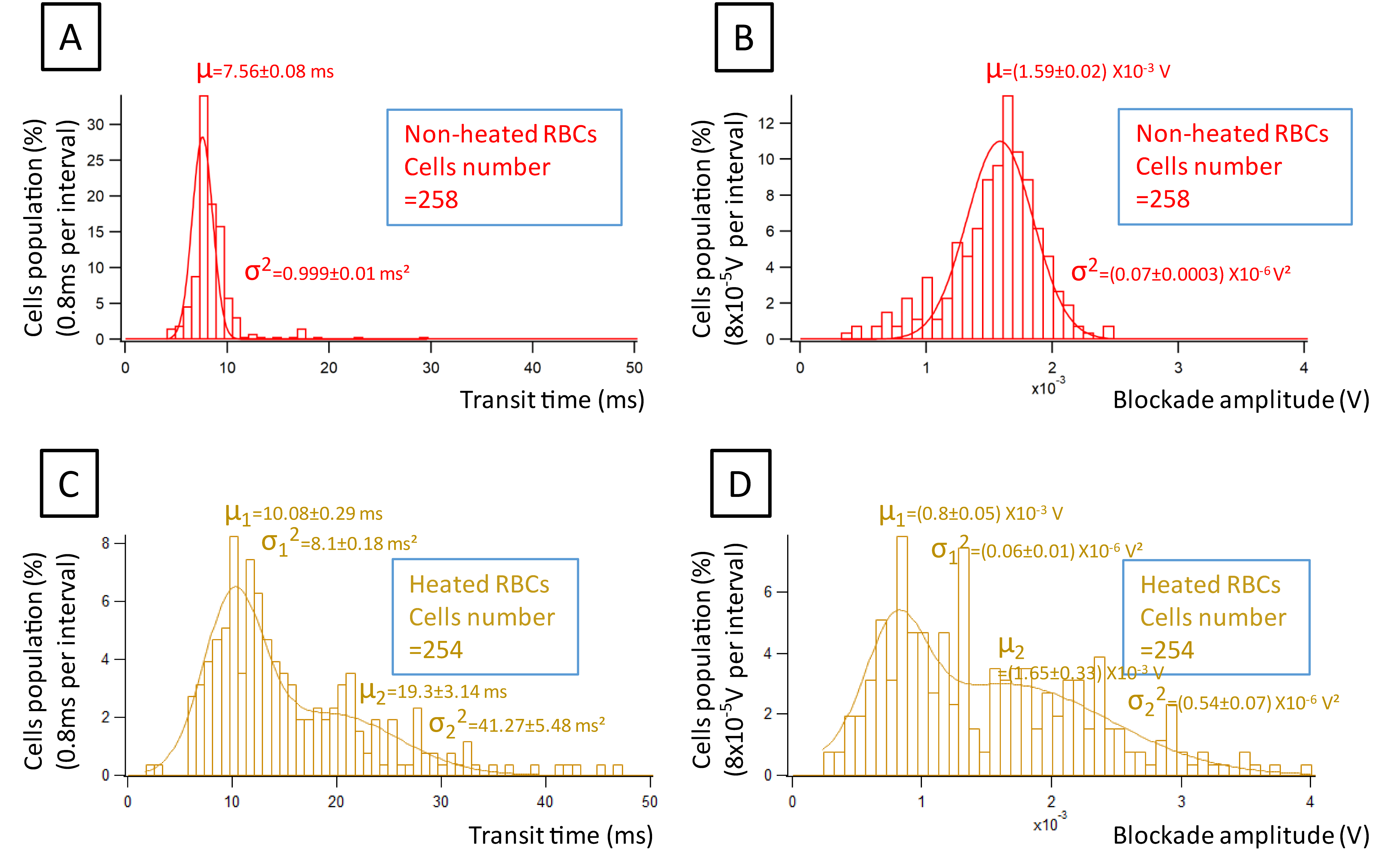

Supplement: Supplementary file 2 — Supplementary information2. [file 41598_2020_66693_MOESM2_ESM.tif]

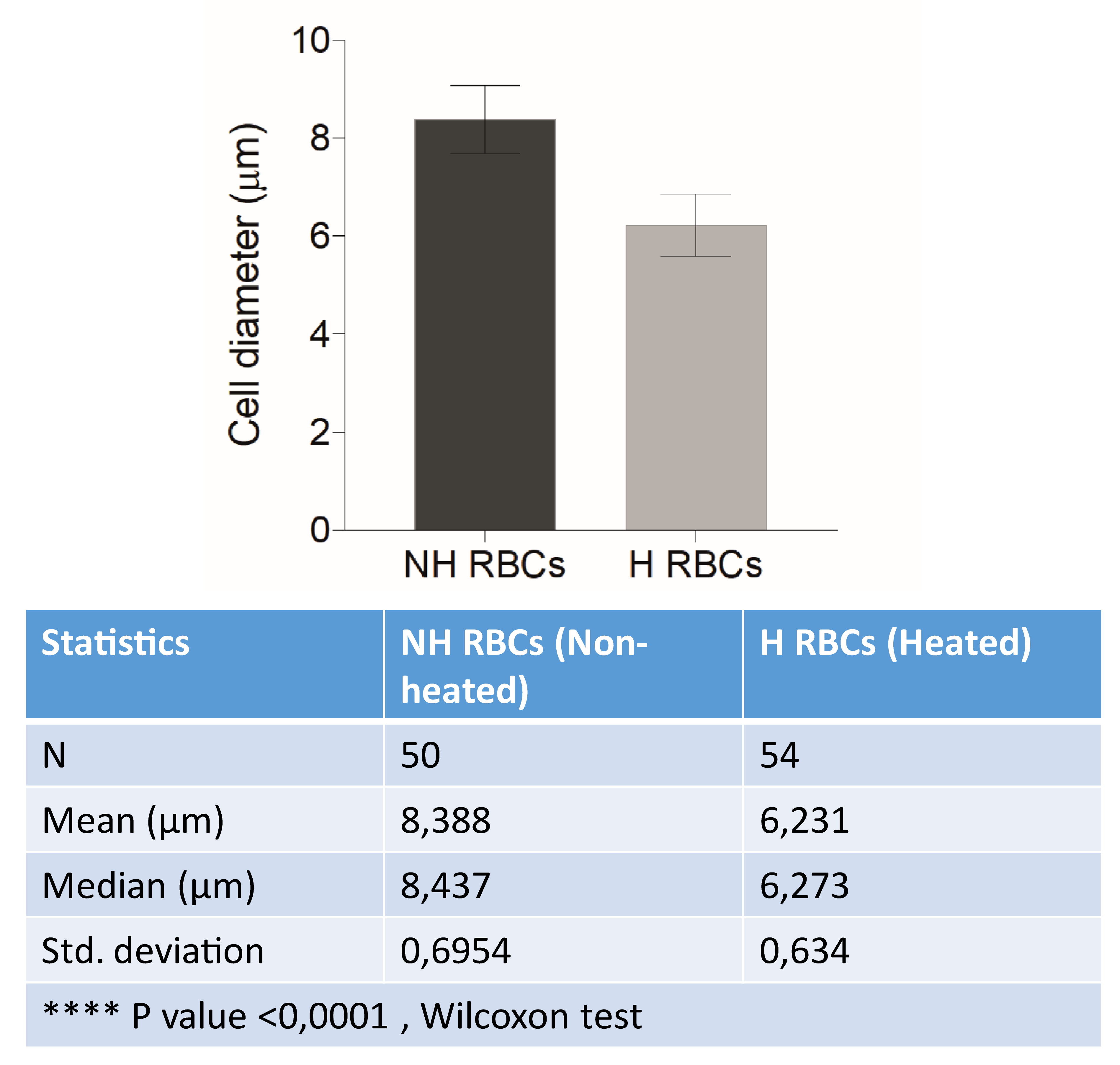

Supplement: Supplementary file 3 — Supplementary information3. [file 41598_2020_66693_MOESM3_ESM.tif]

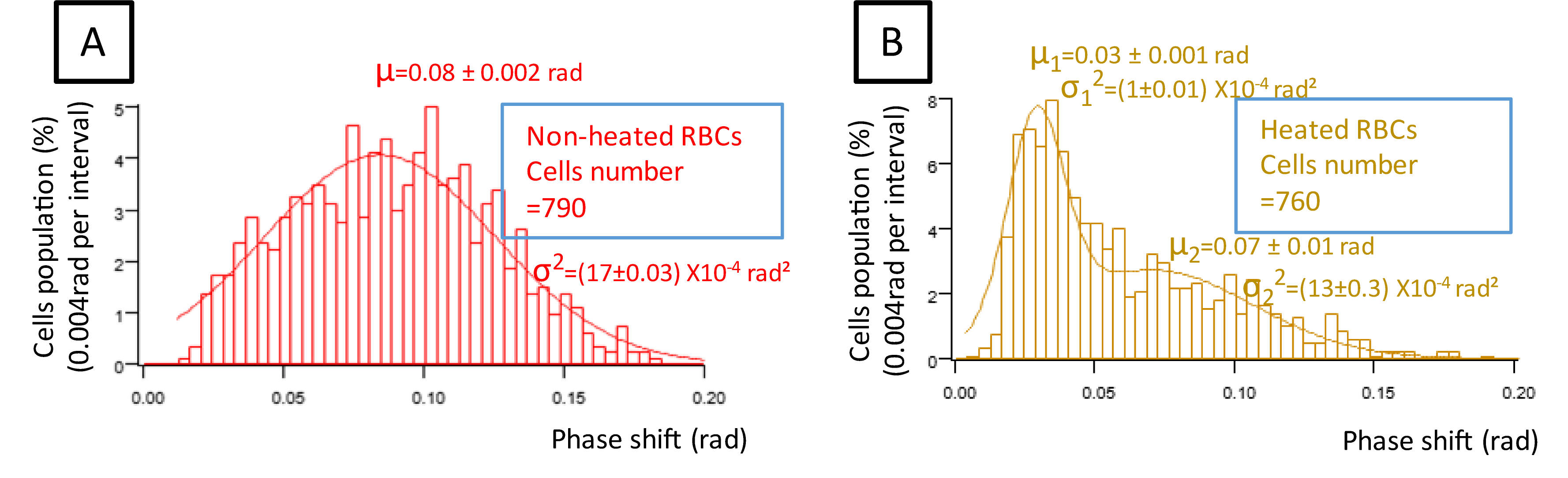

Supplement: Supplementary file 4 — Supplementary information4. [file 41598_2020_66693_MOESM4_ESM.tif]

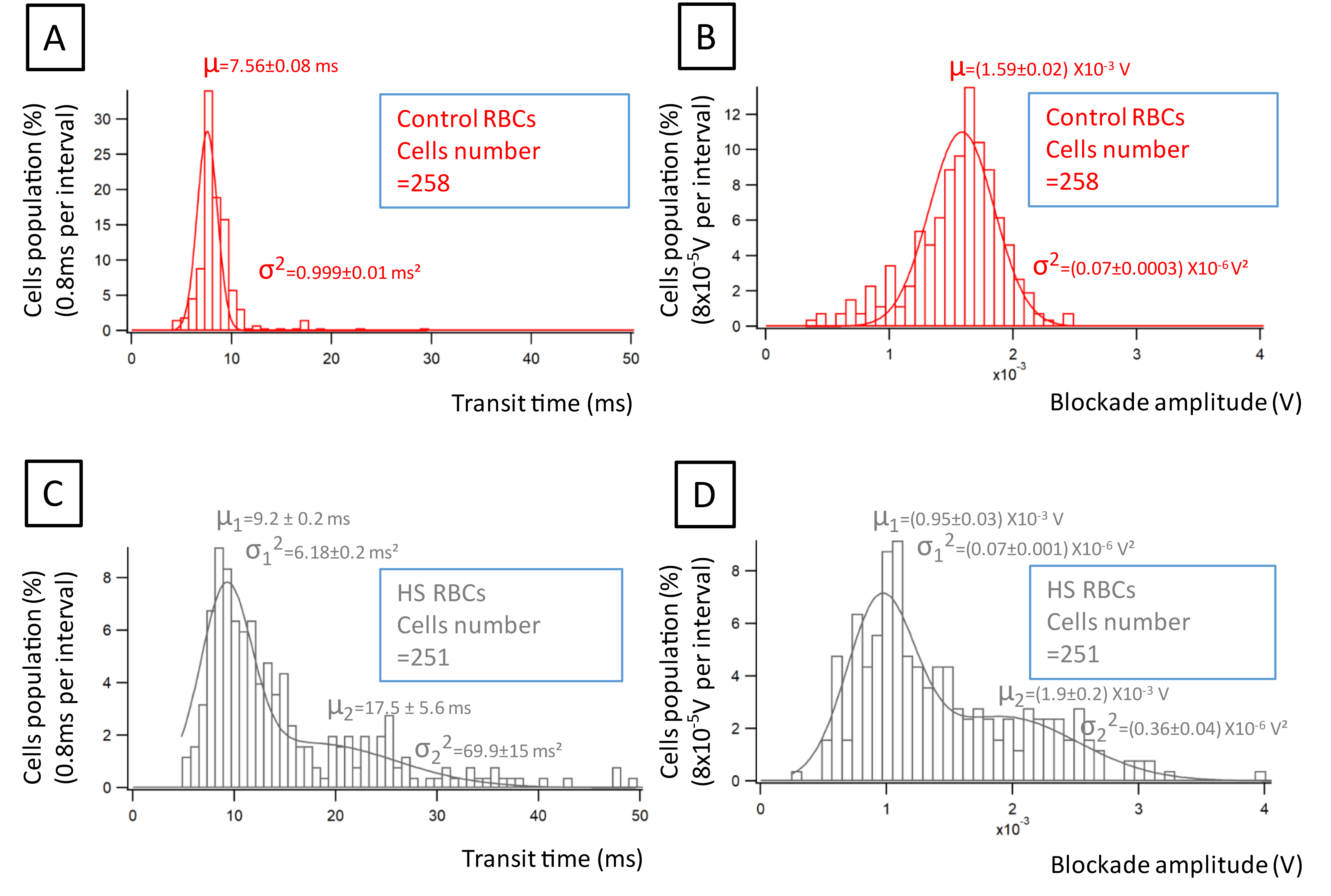

Supplement: Supplementary file 5 — Supplementary information5. [file 41598_2020_66693_MOESM5_ESM.tif]

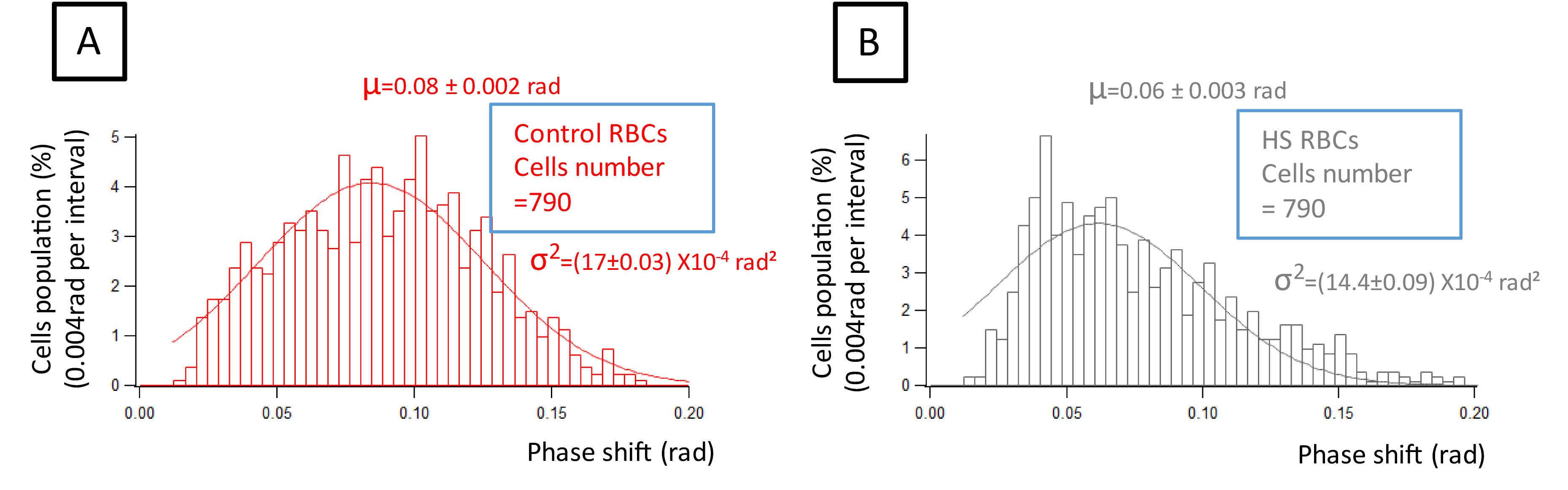

Supplement: Supplementary file 6 — Supplementary information6. [file 41598_2020_66693_MOESM6_ESM.tif]

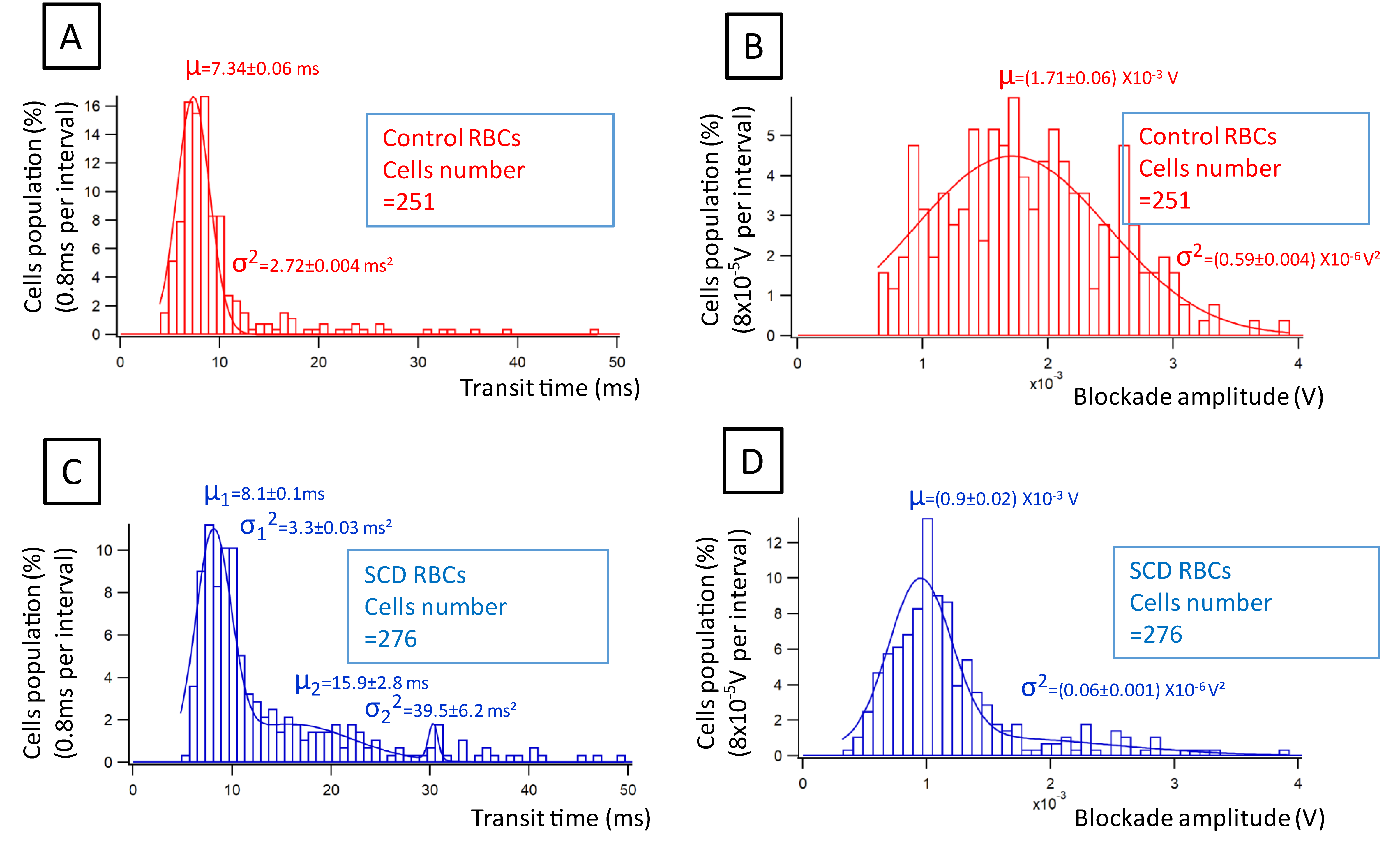

Supplement: Supplementary file 7 — Supplementary information7. [file 41598_2020_66693_MOESM7_ESM.tif]

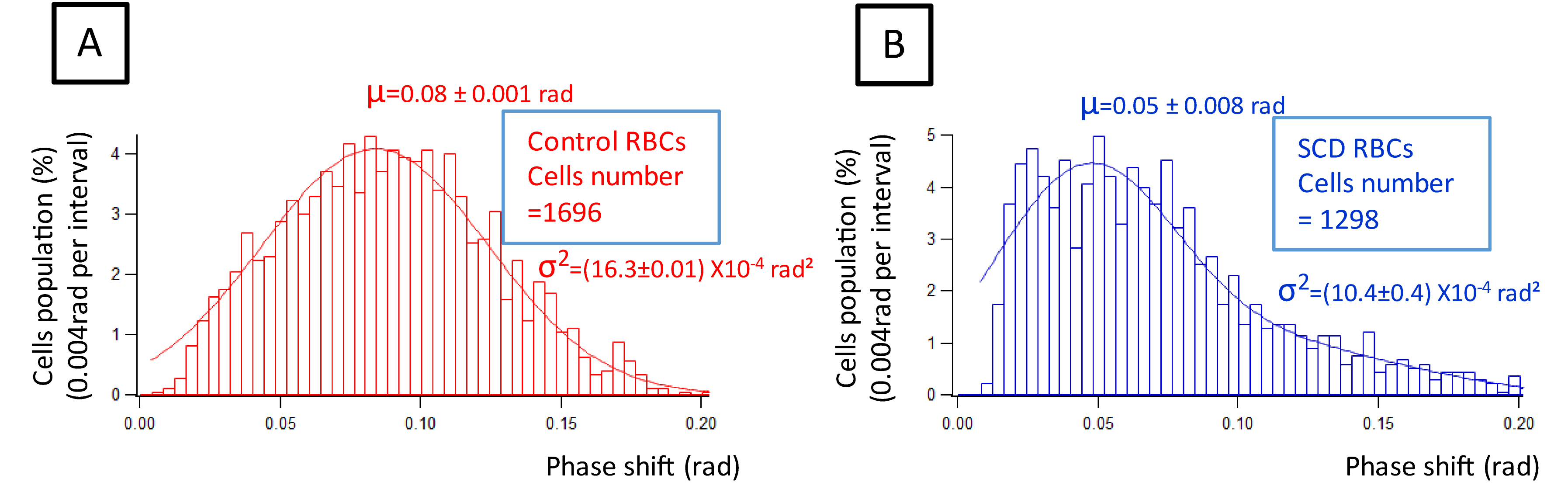

Supplement: Supplementary file 8 — Supplementary information8. [file 41598_2020_66693_MOESM8_ESM.tif]

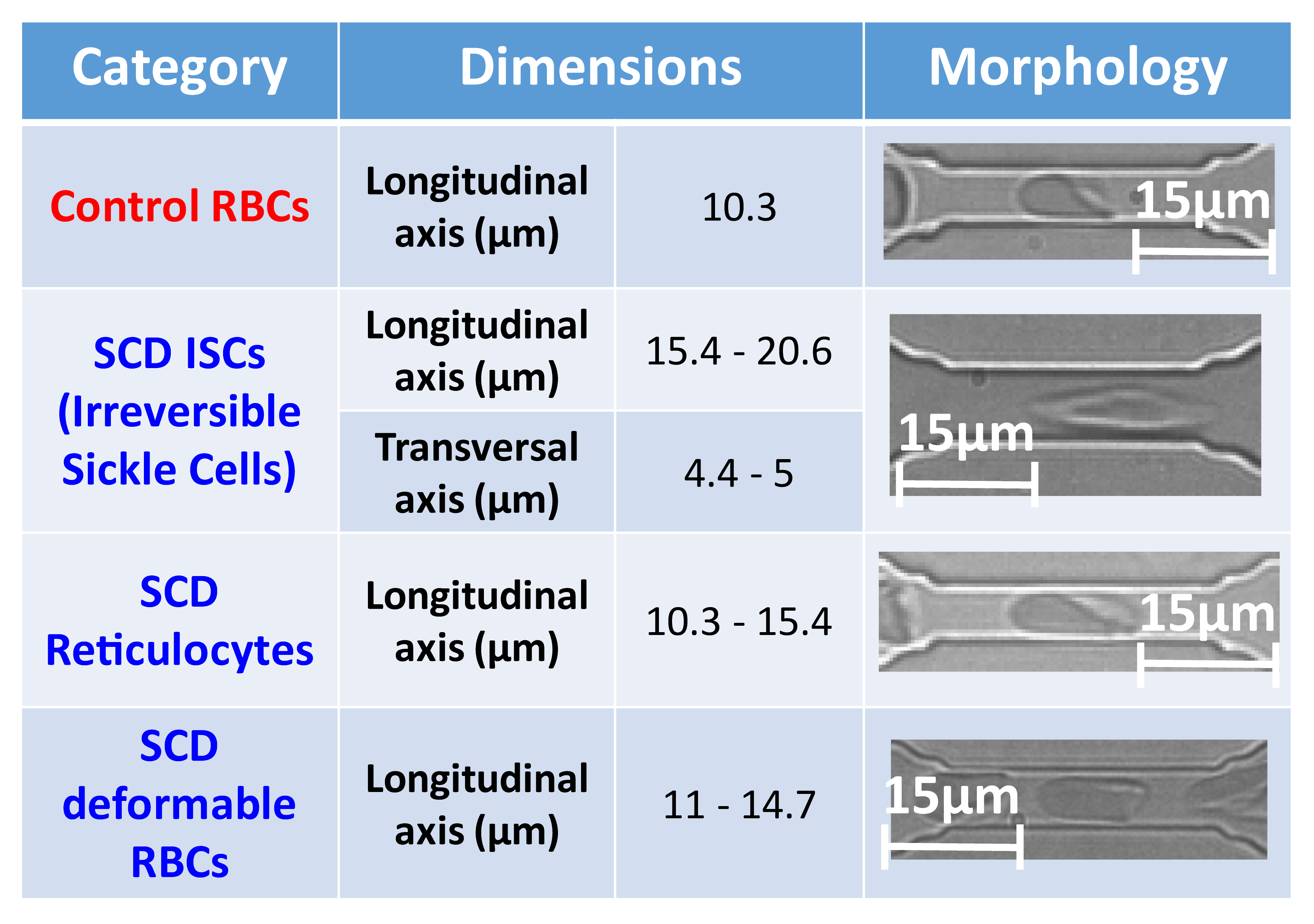

Supplement: Supplementary file 9 — Supplementary information9. [file 41598_2020_66693_MOESM9_ESM.tif]

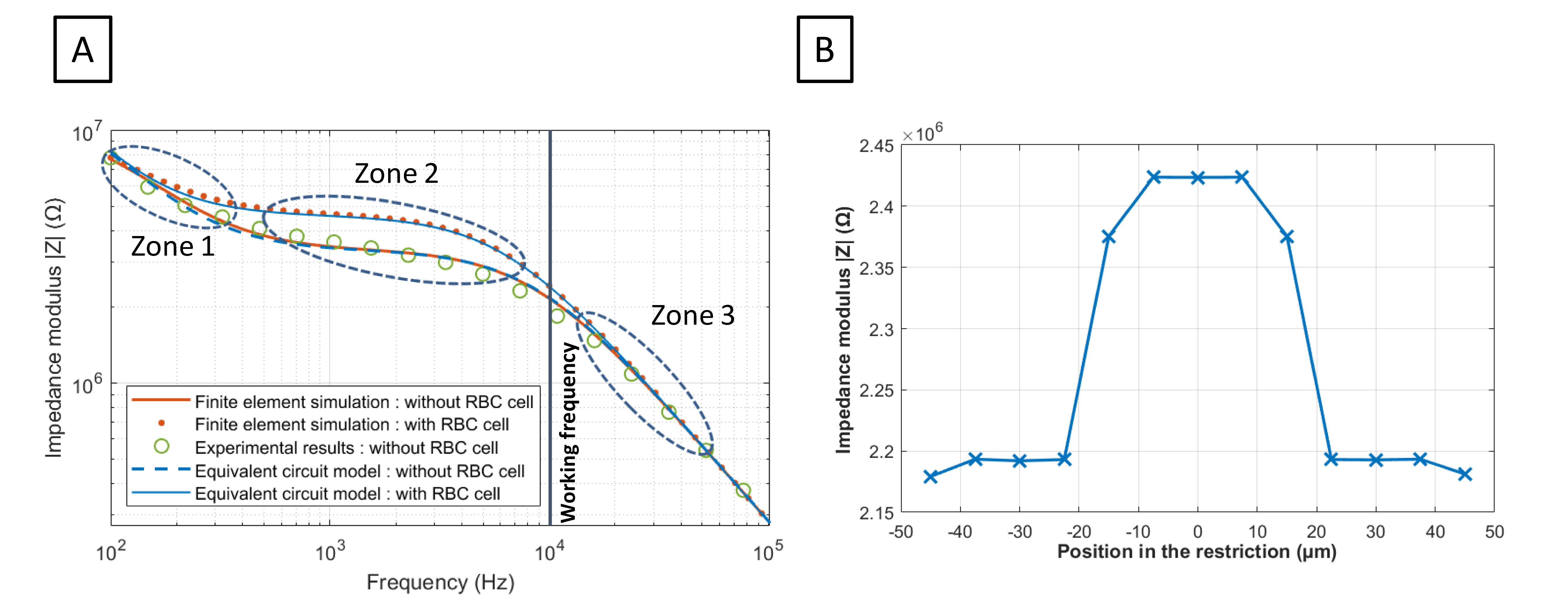

Supplement: Supplementary file 11 — Supplementary information11. [file 41598_2020_66693_MOESM11_ESM.tif]
