# Supplementary material for: Characterization of red blood cell microcirculatory parameters using a bioimpedance microfluidic device
Source: Sci Rep. 2020 Jun 17;10:9869. doi: 10.1038/s41598-020-66693-4 (PMC7299978; doi:10.1038/s41598-020-66693-4)
Supplement: Supplementary file 12 — Supplementary information12. [file 41598_2020_66693_MOESM12_ESM.docx]

**Characterization of red blood cell microcirculatory parameters using a bioimpedance microfluidic device**

**Tieying Xu^1^, Maria A. Lizarralde-Iragorri^2,3,4^, Jean Roman^1^, Rasta Ghasemi^6^, Jean-Pierre Lefèvre^7,10^, Emile Martincic^8^, Valentine Brousse^2,3,4,5^, Olivier Français**^#^**^1,9^, Wassim El Nemer**^#^**^2,3,4^, Bruno Le Pioufle^1,*^**

^1^ Université Paris-Saclay, ENS Paris-Saclay, CNRS, Institut d'Alembert, SATIE, Gif sur Yvette, 91190, France

^2^ Université de Paris, UMR_S1134, BIGR, Inserm, F-75015 Paris, France

^3^ Institut National de Transfusion Sanguine, F-75015 Paris, France

^4^ Laboratoire d’Excellence GR-Ex, F-75013 Paris, France

^5^ Service de Pédiatrie Générale et Maladies Infectieuses, Hôpital Universitaire Necker Enfants Malades, F-75015 Paris, France

^6^ Université Paris-Saclay, ENS Paris-Saclay, CNRS, Institut d’Alembert, Gif sur Yvette, 91190, France

^7^ Université Paris-Saclay, ENS Paris-Saclay, CNRS, PPSM, Institut d’Alembert, Gif sur Yvette, 91190, France

^8^ Centre de Nanosciences et de Nanotechnologies C2N, CNRS, Université Paris-Sud, Université Paris-Saclay, F-91120 Palaiseau, France

^9^ ESYCOM, Univ Gustave Eiffel, CNRS UMR 9007, ESIEE Paris, F-77454 Marne-la-Vallee, France

^10^ CNAM, F-75003 Paris, France

^*^ Corresponding Author :

E-mail: bruno.le-pioufle@ens-paris-saclay.fr. Phone: (+33)14740-7736. Address: 61 Avenue du Président Wilson, 94230 Cachan, France

^#^These authors contributed equally to this work


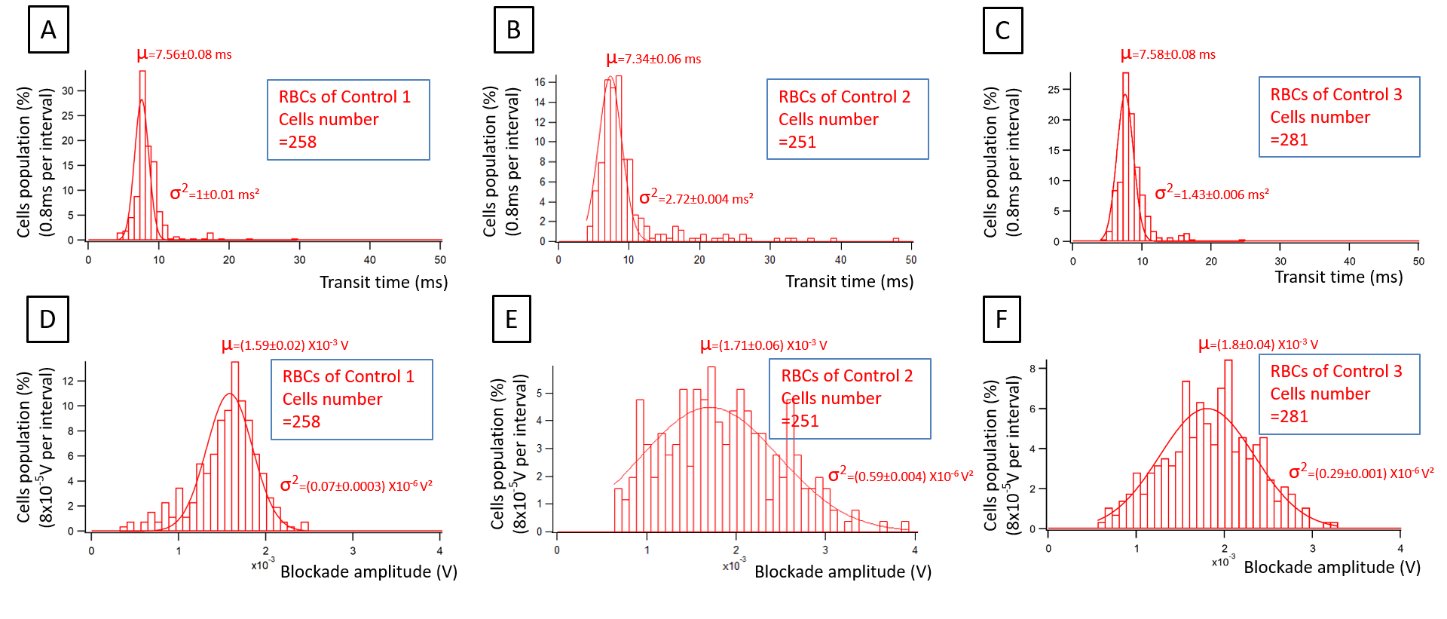


**Supplementary Figure S.1.** Histograms of the transit time and the blockade amplitude for three healthy donor samples. A. Histogram of the transit time for the first healthy donor sample; B. Histogram of the transit time for the second healthy donor sample; C. Histogram of the transit time for the third healthy donor sample; D. Histogram of the blockade amplitude for the first healthy donor sample; E. Histogram of the blockade amplitude for the second healthy donor sample; F. Histogram of the blockade amplitude for the third healthy donor sample.


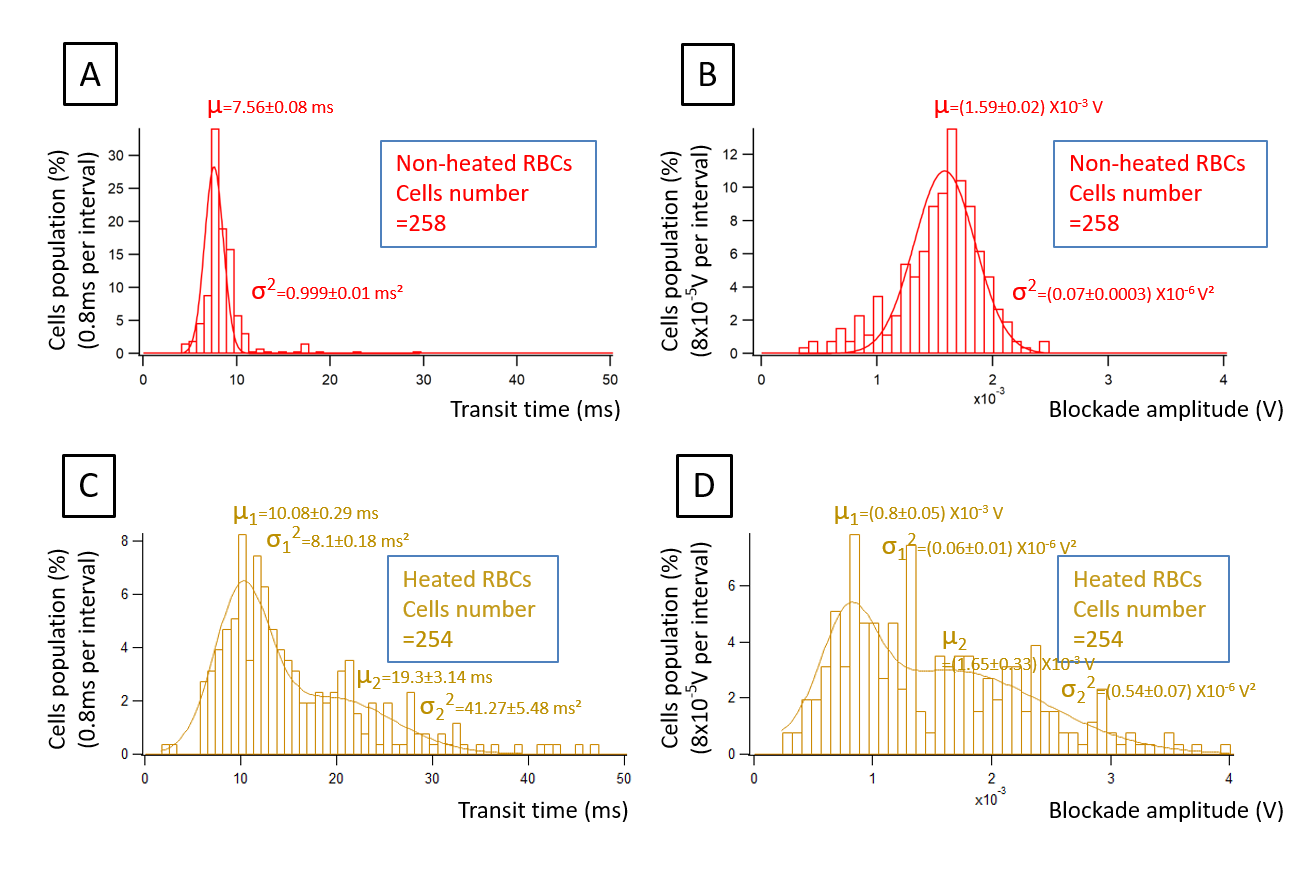


**Supplementary Figure S.2.** Histograms of the transit time and the blockade amplitude for one healthy donor sample. The sample was divided into two parts: Non-heated RBCs and Heated RBCs. A. Histogram of the transit time for non-heated RBCs of one healthy donor sample; B. Histogram of the blockade amplitude for non-heated RBCs of one healthy donor sample; C. Histogram of the transit time for heated RBCs of one healthy donor sample; D. Histogram of the blockade amplitude for heated RBCs of one healthy donor sample.


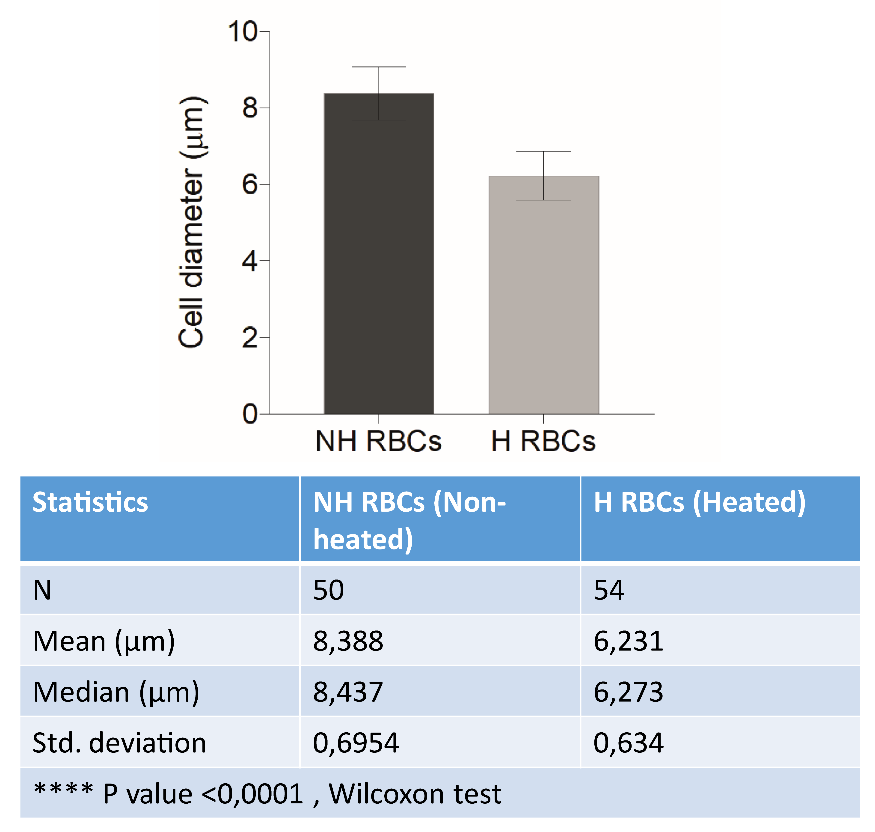


**Supplementary Figure S.3.** Comparison of cell diameter between non-heated RBCs and heated RBCs. The mean diameter and median for 50 non-heated cells equal to 8.4 µm, with a standard deviation of 0.7. The mean diameter of 54 heated cells equal to 6.2 µm and median is around 6.3 µm, with a standard deviation of 0.6.


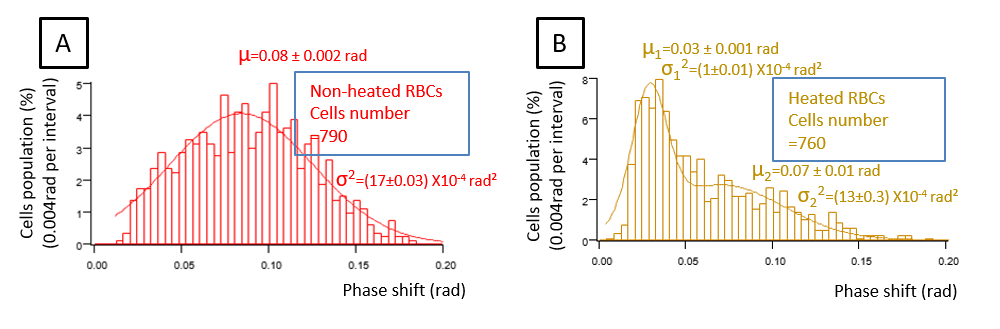
**Supplementary Figure S.4.** Histograms of the phase shift for three healthy donor samples. Each sample was divided into two parts: Non-heated RBCs and Heated RBCs. A decrease of phase shift (0.05 rad) is observed in the case of heated RBCs. A. Histogram of the phase shift for non-heated RBCs of three healthy donor samples; B. Histogram of the phase shift for heated RBCs of three healthy donor samples.


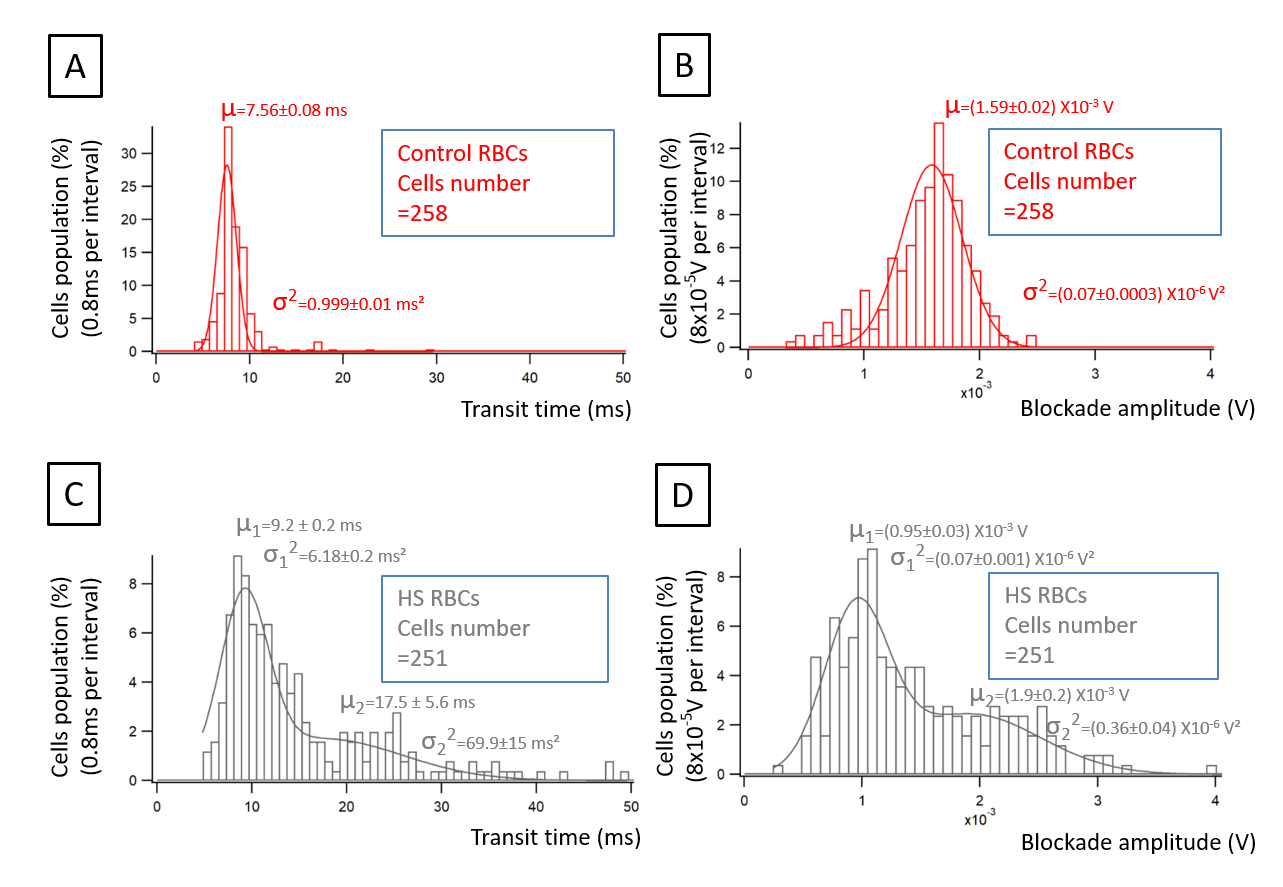


**Supplementary Figure S.5.** Histograms of the transit time and the blockade amplitude for the sample of a hereditary spherocytosis (HS) single patient compared to one healthy donor sample. A. Histogram of the transit time for one healthy donor sample; B. Histogram of the blockade amplitude for one healthy donor sample; C. Histogram of the transit time for the sample of a hereditary spherocytosis (HS) single patient; D. Histogram of the blockade amplitude for the sample of a hereditary spherocytosis (HS) single patient.


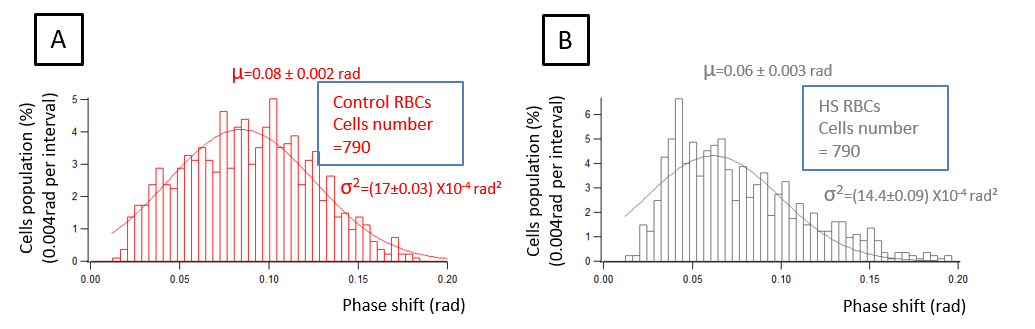


**Supplementary Figure S.6.** Histograms of the phase shift for three hereditary spherocytosis (HS) patient samples compared to three healthy donor samples. A decrease of phase shift equal to 0.02 rad is observed in the case of HS RBCs. A. Histogram of the phase shift for three healthy donor samples; B. Histogram of the phase shift for three hereditary spherocytosis (HS) patient samples.


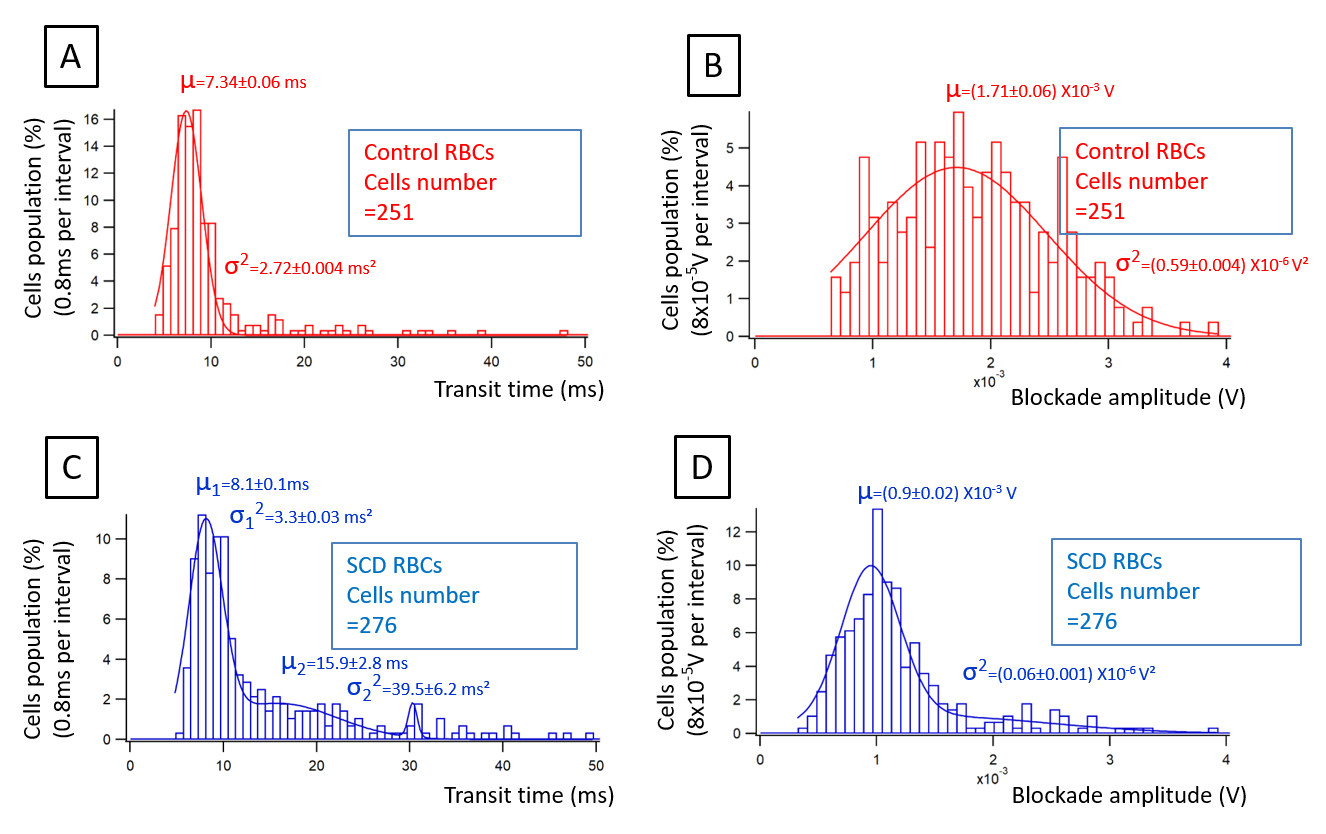


**Supplementary Figure S.7.** Histograms of the transit time and the blockade amplitude for a sickle cell disease (SCD) single patient sample, compared to one healthy donor sample. A. Histogram of the transit time for one healthy donor sample; B. Histogram of the blockade amplitude for one healthy donor sample; C. Histogram of the transit time for a sickle cell disease (SCD) single patient sample; D. Histogram of the blockade amplitude for a sickle cell disease (SCD) single patient sample.


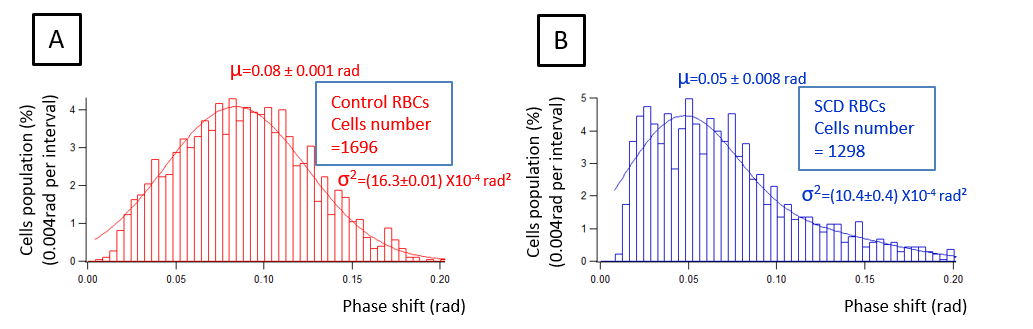


**Supplementary Figure S.8.** Histograms of the phase shift for five sickle cell disease (SCD) patient samples compared to six healthy donor samples. A decrease of phase shift equal to 0.03 rad is observed in the case of SCD RBCs. A. Histogram of the phase shift for six healthy donor samples; B. Histogram of the phase shift for five sickle cell disease (SCD) patient samples.


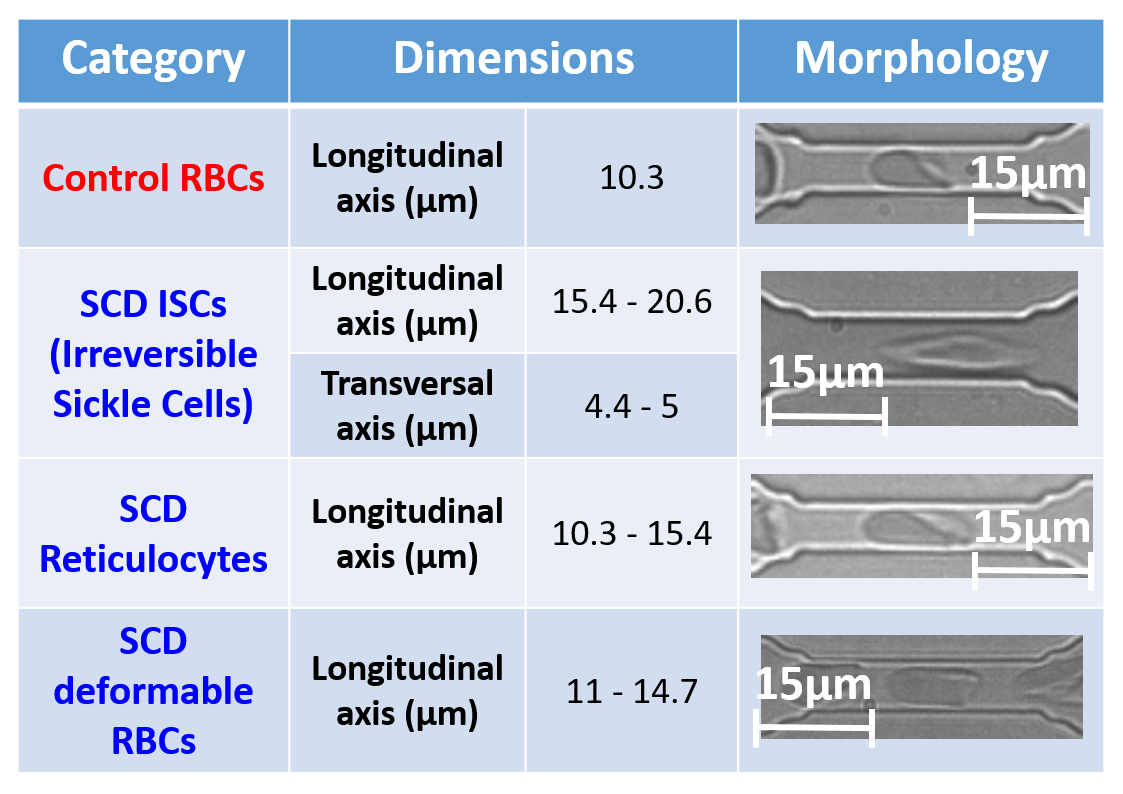


**Supplementary Figure S.9.** Cell dimensions and the corresponding morphology of Control RBCs or different kind of SCD RBCs in the restriction with the width of 5 µm, the height of 2 µm and the length of 30 µm.


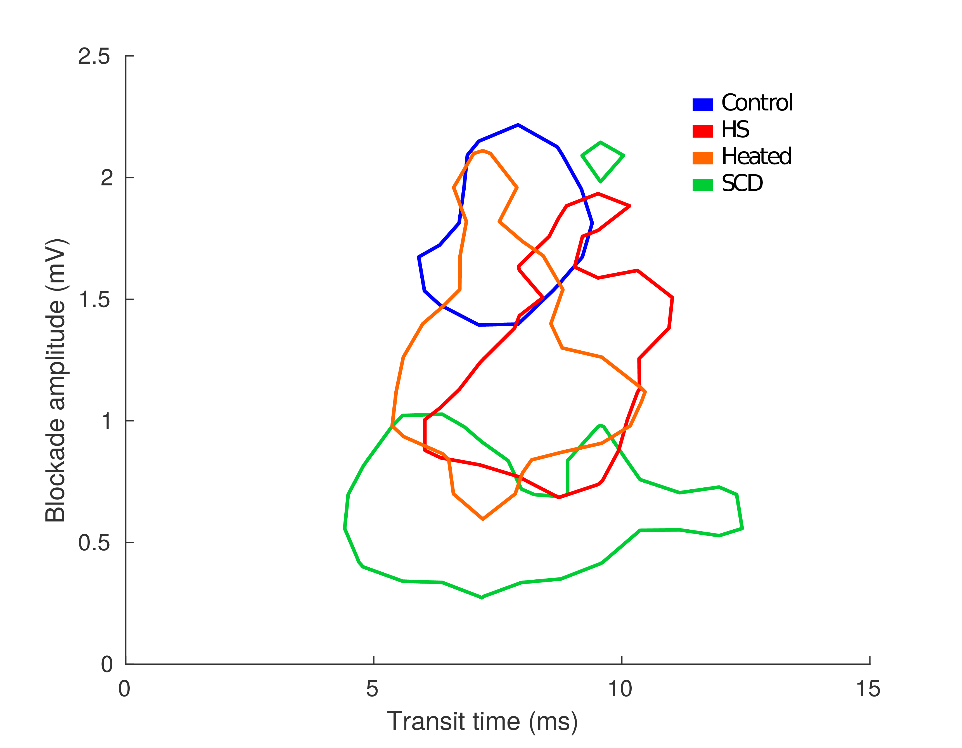


**Supplementary Figure S.10.** Graph representing the blockade amplitude versus the transit time, for different status of the cell. Lines represent equi-levels of density of probability for each type of cell (more than 30% density of probability). The graph is represented for control RBCs (1696 cells), heated RBCs (760 cells) HS RBCs (790 cells) and SCD cells (1298 cells).


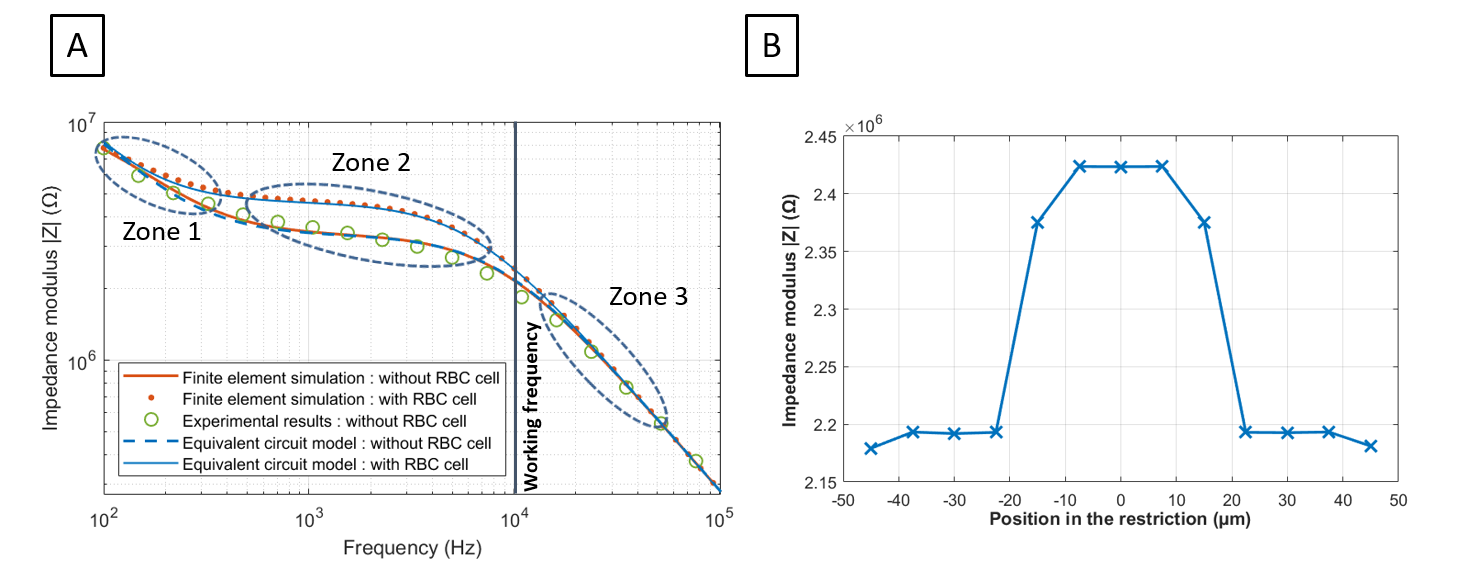


**Supplementary Figure S.11.** A. Simulation results for the bioimpedance spectrum of two cases: without and with RBC in the restriction. There are 3 zones; zone 1: double layer capacity effect due to the electrode polarisation (f<500Hz); zone 2: medium conduction is dominant (500Hz<f<10^4^Hz); zone 3: effect of the low-pass filter of the device due to parasite capacitance (f>10^4^Hz). 10^4^Hz was chosen as the working frequency. B. The bioimpedance evolution for RBC passing through the restriction.

|  | | µ | | σ^2^ |
| --- | --- | --- | --- | --- |
| Non-heated RBCs | Transit time | 7.39±0.09 ms | | 1.69±0.008 ms² |
|  | Blockade amplitude | (1.73±0.02) X10^-3^ V | | (0.27±0.0005) X10^-6^ V² |
| Heated RBCs | Transit time | t_h1_ | 7.95±0.18 ms | 6.7±0.12 ms² |
|  |  | t_h2_ | 16.5±3.17 ms | 49.56±5.06 ms² |
|  | Blockade amplitude | A_h1_ | (0.6±0.02) X10^-3^ V | (0.06±0.0005) X10^-6^ V² |
|  |  | A_h2_ | (1.96±0.12) X10^-3^ V | (0.65±0.02) X10^-6^ V² |

**Supplementary Table S.1.** Expected values µ and variances σ^2^ corresponding to assay presented in Fig. 1.

|  | | µ | | σ^2^ |
| --- | --- | --- | --- | --- |
| Control | Transit time | 7.39±0.11 ms | | 1.69±0.01 ms² |
|  | Blockade amplitude | (1.73±0.02) X10^-3^ V | | (0.27±0.001) X10^-6^ V² |
| HS RBCs | Transit time | t_HS1_ | 8.25 ± 0.09 ms | 1.55±0.02 ms² |
|  |  | t_HS2_ | 12.16 ± 0.54 ms | 19.07±0.12 ms² |
|  | Blockade amplitude | A_HS1_ | (0.92±0.02) X10^-3^ V | (0.02±0.001) X10^-6^ V² |
|  |  | A_HS2_ | (1.38±0.07) X10^-3^ V | (0.32±0.004) X10^-6^ V² |
|  |  | A_HS3_ | (2.46±0.06) X10^-3^ V | (0.06±0.004) X10^-6^ V² |

Supplementary Table S.2. Expected values µ and variances σ^2^ corresponding to assay presented in Fig. 2.

|  | | µ | | σ^2^ |
| --- | --- | --- | --- | --- |
| Control | Transit time | 7.13±0.09 ms | | 3.08±0.01 ms² |
|  | Blockade amplitude | (1.76±0.01) X10^-3^ V | | (0.26±0.0002) X10^-6^ V² |
| SCD RBCs | Transit time | t_SCD1_ | 7.54±0.1 ms | 1.12±0.03 ms² |
|  |  | t_SCD2_ | 13.26±2.21 ms | 1.99±0.02 ms² |
|  | Blockade amplitude | A_SCD1_ | (1.12±0.03) X10^-3^ V | (0.13±0.01) X10^-6^ V² |
|  |  | A_SCD2_ | (1.99±0.02) X10^-3^ V | (0.01±0.001) X10^-6^ V² |
|  |  | A_SCD3_ | (2.09±0.46) X10^-3^ V | (0.73±0.06) X10^-6^ V² |

**Supplementary Table S.3.** Expected values µ and variances σ^2^ corresponding to assay presented in Fig. 3.
